# *WNT16* Influences Bone Mineral Density, Cortical Bone Thickness, Bone Strength, and Osteoporotic Fracture Risk

**DOI:** 10.1371/journal.pgen.1002745

**Published:** 2012-07-05

**Authors:** Hou-Feng Zheng, Jon H. Tobias, Emma Duncan, David M. Evans, Joel Eriksson, Lavinia Paternoster, Laura M. Yerges-Armstrong, Terho Lehtimäki, Ulrica Bergström, Mika Kähönen, Paul J. Leo, Olli Raitakari, Marika Laaksonen, Geoffrey C. Nicholson, Jorma Viikari, Martin Ladouceur, Leo-Pekka Lyytikäinen, Carolina Medina-Gomez, Fernando Rivadeneira, Richard L. Prince, Harri Sievanen, William D. Leslie, Dan Mellström, John A. Eisman, Sofia Movérare-Skrtic, David Goltzman, David A. Hanley, Graeme Jones, Beate St. Pourcain, Yongjun Xiao, Nicholas J. Timpson, George Davey Smith, Ian R. Reid, Susan M. Ring, Philip N. Sambrook, Magnus Karlsson, Elaine M. Dennison, John P. Kemp, Patrick Danoy, Adrian Sayers, Scott G. Wilson, Maria Nethander, Eugene McCloskey, Liesbeth Vandenput, Richard Eastell, Jeff Liu, Tim Spector, Braxton D. Mitchell, Elizabeth A. Streeten, Robert Brommage, Ulrika Pettersson-Kymmer, Matthew A. Brown, Claes Ohlsson, J. Brent Richards, Mattias Lorentzon

**Affiliations:** 1 Department of Medicine, Human Genetics, McGill University, Montreal, Canada; 2 Department of Epidemiology and Biostatistics, Lady Davis Institute for Medical Research, Jewish General Hospital, Montreal, Canada; 3 Musculoskeletal Research Unit, School of Clinical Sciences, University of Bristol, Bristol, United Kingdom; 4 Human Genetics Group, University of Queensland Diamantina Institute, Princess Alexandra Hospital, University of Queensland, Brisbane, Australia; 5 Endocrinology, Royal Brisbane and Women's Hospital, Brisbane, Australia; 6 Medical Research Council Centre for Causal Analyses in Translational Epidemiology, University of Bristol, Bristol, United Kingdom; 7 School of Social and Community Medicine, University of Bristol, Bristol, United Kingdom; 8 Center for Bone and Arthritis Research, Institute of Medicine, Sahlgrenska Academy, University of Gothenburg, Gothenburg, Sweden; 9 Department of Medicine, Division of Endocrinology, Diabetes, and Nutrition, University of Maryland School of Medicine, Baltimore, Maryland, United States of America; 10 Department of Clinical Chemistry, Fimlab, University of Tampere School of Medicine and Tampere University Hospital, Tampere, Finland; 11 Surgical and Perioperative Sciences, Umeå University, Umeå, Sweden; 12 Department of Clinical Physiology, University of Tampere School of Medicine and Tampere University Hospital, Tampere, Finland; 13 Research Centre of Applied and Preventive Cardiovascular Medicine and the Department of Clinical Physiology and Nuclear Medicine, University of Turku and Turku University Hospital, Turku, Finland; 14 Department of Food and Environmental Sciences, University of Helsinki, Helsinki, Finland; 15 Rural Clinical School, The University of Queensland, Toowoomba, Australia; 16 Department of Medicine, University of Turku and Turku University Hospital, Turku, Finland; 17 Montreal Heart Institute, Research Institute, Montreal, Canada; 18 Department of Internal Medicine, Erasmus University Medical Center, Rotterdam, The Netherlands; 19 Department of Epidemiology, Erasmus University Medical Center, Rotterdam, The Netherlands; 20 Endocrinology and Diabetes, Sir Charles Gairdner Hospital, Perth, Australia; 21 School of Medicine and Pharmacology, University of Western Australia, Perth, Australia; 22 Bone Research Group, UKK Institute, Tampere, Finland; 23 Department of Internal Medicine, University of Manitoba, Winnipeg, Canada; 24 Garvan Institute of Medical Research, University of New South Wales, Sydney, Australia; 25 Department of Medicine, McGill University, Montreal, Canada; 26 Department of Medicine, University of Calgary, Calgary, Canada; 27 Menzies Research Institute, University of Tasmania, Hobart, Australia; 28 Centre for Bone and Periodontal Research, McGill University, Montreal, Canada; 29 Department of Medicine, University of Auckland, Auckland, New Zealand; 30 Kolling Institute, Royal North Shore Hospital, University of Sydney, Sydney, Australia; 31 Clinical and Molecular Osteoporosis Research Unit, Department of Orthopaedics, Skane University Hospital, Lund University, Malmö, Sweden; 32 Medical Research Council Lifecourse Epidemiology Unit, University of Southampton, Southampton, United Kingdom; 33 Twin Research and Genetic Epidemiology, King's College London, London, United Kingdom; 34 Genomics Core Facility, University of Gothenburg, Gothenburg, Sweden; 35 Academic Unit of Bone Metabolism, Metabolic Bone Centre, University of Sheffield, Sheffield, United Kingdom; 36 NIHR Musculoskeletal Biomedical Research Unit, Sheffield Teaching Hospitals, Sheffield, United Kingdom; 37 Lexicon Pharmaceuticals, The Woodlands, Texas, United States of America; 38 Geriatric Research and Education Clinical Center (GRECC), Veterans Administration Medical Center, Baltimore, Maryland, United States of America; 39 Department of Pharmacology and Neuroscience, Umeå University, Umeå, Sweden; 40 Department of Public Health and Clinical Medicine, Umeå Unviersity, Umeå, Sweden; Georgia Institute of Technology, United States of America

## Abstract

We aimed to identify genetic variants associated with cortical bone thickness (CBT) and bone mineral density (BMD) by performing two separate genome-wide association study (GWAS) meta-analyses for CBT in 3 cohorts comprising 5,878 European subjects and for BMD in 5 cohorts comprising 5,672 individuals. We then assessed selected single-nucleotide polymorphisms (SNPs) for osteoporotic fracture in 2,023 cases and 3,740 controls. Association with CBT and forearm BMD was tested for ∼2.5 million SNPs in each cohort separately, and results were meta-analyzed using fixed effect meta-analysis. We identified a missense SNP (Thr>Ile; rs2707466) located in the *WNT16* gene (7q31), associated with CBT (effect size of −0.11 standard deviations [SD] per C allele, P = 6.2×10^−9^). This SNP, as well as another nonsynonymous SNP rs2908004 (Gly>Arg), also had genome-wide significant association with forearm BMD (−0.14 SD per C allele, P = 2.3×10^−12^, and −0.16 SD per G allele, P = 1.2×10^−15^, respectively). Four genome-wide significant SNPs arising from BMD meta-analysis were tested for association with forearm fracture. SNP rs7776725 in *FAM3C*, a gene adjacent to *WNT16*, was associated with a genome-wide significant increased risk of forearm fracture (OR = 1.33, P = 7.3×10^−9^), with genome-wide suggestive signals from the two missense variants in *WNT16* (rs2908004: OR = 1.22, P = 4.9×10^−6^ and rs2707466: OR = 1.22, P = 7.2×10^−6^). We next generated a homozygous mouse with targeted disruption of *Wnt16*. Female *Wnt16^−/−^* mice had 27% (P<0.001) thinner cortical bones at the femur midshaft, and bone strength measures were reduced between 43%–61% (6.5×10^−13^<P<5.9×10^−4^) at both femur and tibia, compared with their wild-type littermates. Natural variation in humans and targeted disruption in mice demonstrate that *WNT16* is an important determinant of CBT, BMD, bone strength, and risk of fracture.

## Author Summary

Bone traits are highly dependent on genetic factors. To date, numerous genetic loci for bone mineral density (BMD) and only one locus for osteoporotic fracture have been previously identified to be genome-wide significant. Cortical bone has been reported to be an important determinant of bone strength; so far, no genome-wide association studies (GWAS) have been performed for cortical bone thickness (CBT) of the tibial and radial diaphysis or BMD at forearm, a skeletal site rich in cortical bone. Therefore, we performed two separated meta-analyses of GWAS for cortical thickness of the tibia in 3 independent cohorts of 5,878 men and women, and for forearm BMD in 5 cohorts of 5,672 individuals. We identified the 7q31 locus, which contains *WNT16*, to be associated with CBT and BMD. Four SNPs from this locus were then tested in 2,023 osteoporotic fracture cases and 3,740 controls. One of these SNPs was genome-wide significant, and two were genome-wide suggestive, for forearm fracture. Generating a mouse with targeted disruption of *Wnt16*, we also demonstrated that mice lacking this protein had substantially thinner bone cortices and reduced bone strength than their wild-type littermates. These findings highlight *WNT16* as a clinically relevant member of the Wnt signaling pathway and increase our understanding of the etiology of osteoporosis-related phenotypes and fracture.

## Introduction

Osteoporosis is a common skeletal disease characterized by reduced areal bone mineral density (BMD) and defects in the microarchitecture of bone, resulting in an increased risk of fragility fracture [1]. Osteoporotic fractures affect between one third to one half of white women [2] and currently incur direct costs exceeding $19 billion per year in the United States alone [3]; and this socio-economic burden is increasing with the ageing of industrial societies [4].

Twin and family studies have revealed that genetic factors can explain up to 85% of the variation in peak BMD [5], [6]. Since 2007, we and others have published several genome-wide association studies (GWAS) for osteoporosis and related traits [7], [8], [9], [10], [11], [12], [13], [14] identifying multiple common variants associated with BMD and highlighting biologic pathways that influence BMD.

Most osteoporotic fractures occur at peripheral sites, mainly containing cortical bone, after the age of 65 [15]. As indicated by a recent study, bone loss at this age is mainly due to loss in cortical and not trabecular bone [16]. In human cadaver femurs, cortical bone has been reported to be the main determinant of the femoral neck bone strength, while trabecular bone only contributes marginally to bone strength at this site [17]. Evidence implicating cortical thinning as a risk factor for hip fracture has also been presented [18]. The heritability for cortical thickness, measured using computed tomography, has been reported to be as high as 51% [19].

BMD is a complex trait, obtained from a 2-dimensional projectional scan of the given bone with dual x-ray absorptiometry (DXA). Although BMD is the most clinical useful measure for diagnosing bone fragility (osteoporosis), it fails to provide a detailed skeletal phenotype necessary to discern traits such as bone geometry and volumetric BMD (vBMD) [20]. Most of the loci or genes identified have been associated with BMD at lumbar spine and/or femoral neck, sites rich in trabecular bone. Therefore, we hypothesized that investigating BMD at the forearm, a primarily cortical bone site, as well cortical bone thickness, a trait with high heritability, would serve as successful strategies to identify novel bone related genetic loci.

Forearm fractures are among the most common fractures, affecting 1.7 million individuals per year. In contrast to hip fractures [21], forearm fractures have been shown to be highly heritable, with estimates of 54% [22]. To our knowledge, no GWA studies for cortical bone thickness, forearm BMD or fractures have been published. Importantly, we are aware of only one previous locus [12] that has been associated with risk of fracture even in large-scale meta-analytic efforts at a genome-wide significant level (reviewed previously) [23], [24], [25].

In this study, we performed two separate GWA meta-analyses in order to identify loci for cortical bone thickness of tibial diaphysis and BMD at the distal radius. Firstly, we performed a GWA study of three large and well-characterized independent discovery cohorts of 5,878 samples with the aim of identifying genetic loci for cortical thickness. SNPs meeting GWAS significance in the discovery meta-analysis were also tested for association in a large replication cohort (N = 1032). In the second and separate GWA meta-analysis, we combined genome-wide association results of 5,672 samples with BMD measurement at the forearm site from five cohorts; we then sought evidence of association of selected genome-wide significant signals in three cohorts comprising 5,763 individuals for forearm fracture.

To determine the possible functional role of the identified genes on cortical bone thickness and bone strength, we generated mice with inactivated genes and investigated their skeletal phenotype. The resultant findings increase our understanding of the genetic basis of osteoporosis and osteoporotic fracture.

## Results

### GWAS Meta-Analysis of Cortical Thickness

**Figure 1:**
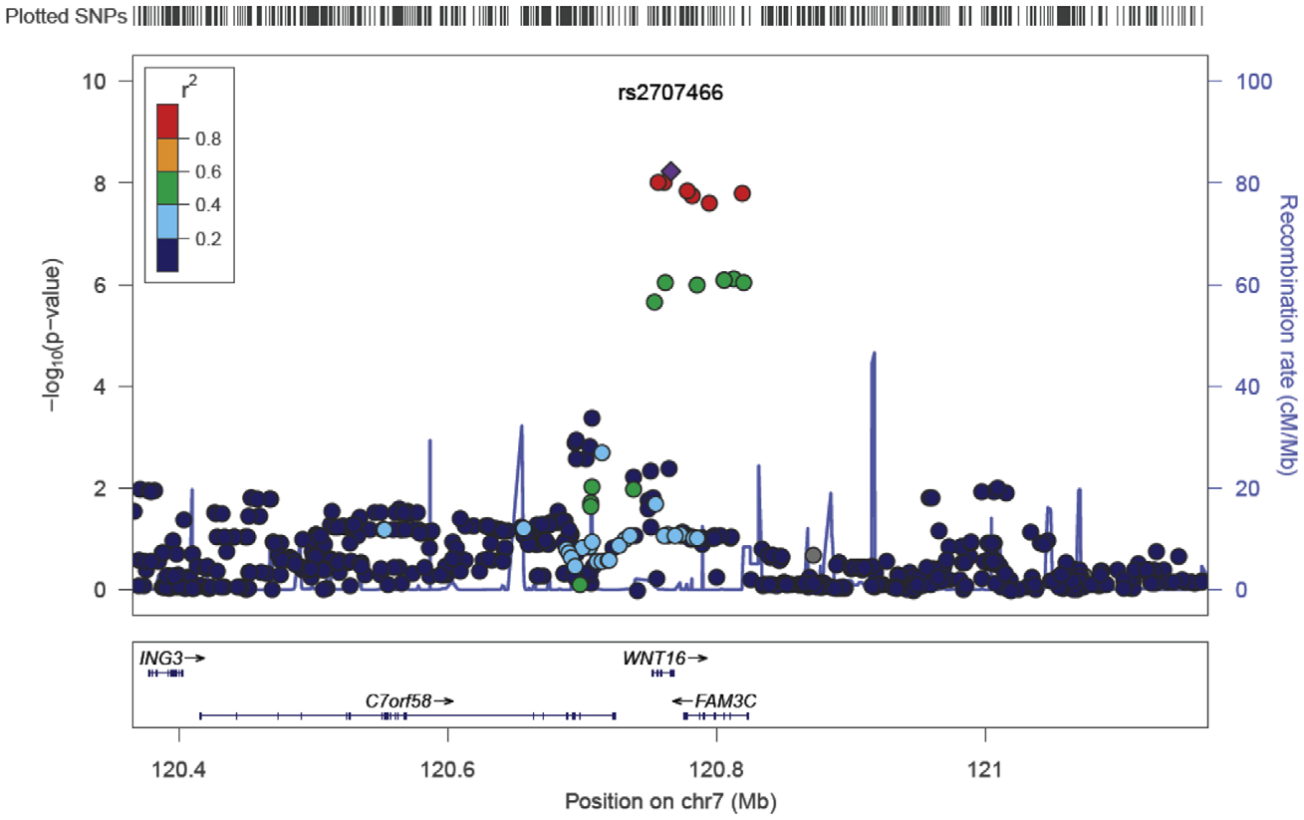
SNP rs2707466 regional association plot of the discovery genome-wide meta-analysis of cortical thickness. Circles show GWA meta-analysis p-values, with different colors indicating varying linkage disequilibrium with rs2707466 (diamond).

Anthropometrics, and bone variables for the three discovery GWAS cohorts and one replication cohort are presented in Table S1. Marked deviation from the null distribution amongst the lowest observed p-values were observed for the meta-analysis results (Figure S1). The results showed that the greatest evidence for association between genetic variation and tibial cortical thickness was seen for rs9525638 on chromosome 13, slightly upstream of the *RANKL* gene (−0.11 standard deviations [SD] per T allele, P = 3.3×10^−10^) (Table 1, Figure S2 and Figure S3). The second strongest genetic signal (rs2707466) for cortical thickness was located at the *WNT16* locus (−0.10 SD per C allele, P = 5.9×10^−9^) (Table 1, Figure 1 and Figure S2). The SNP rs2707466 represents a missense polymorphism (Thr>Ile) located in the fourth exon of *WNT16*.

**Table 1:** Top cortical thickness GWA meta-analysis hits, with replication and meta-analysis of all four cohorts.

			Discovery									Meta-Analysis				Replication			Combined		
			Alspac discovery			GOOD discovery			YFS Discovery							MrOs Sweden			All cohorts		
Gene/Position	SNP	Effect allele	N	Beta (se)	p	N	Beta (se)	p	N	Beta (se)	p	Allele freq	N	Beta (se)	p	N	Beta (se)	p	N	Beta (se)	p
*TNFSF11*	rs9525638	T	3382	−0.11	2.1E−07	938	−0.08	0.06	1558	−0.11	2.1E−03	0.54	5878	−0.11	3.3E−10	1021	−0.02	0.74	6899	−0.09	3.6E−9
13: 42026577			(0.02)			(0.04)			(0.04)				(0.02)		(0.04)			(0.02)			
*WNT16*	rs2707466	C	3382	−0.08	1.6E−04	938	−0.14	2.2E−03	1558	−0.14	1.7E−04	0.58	5878	−0.10	5.9E−9	1032	−0.11	8.0E−03	6910	−0.11	1.5E−10
7: 120766325				(0.02)			(0.05)			(0.04)				(0.02)		(0.04)			(0.02)		

Models adjusted for sex (ALSPAC and YFS), age, height, weight (ln). Betas in standard deviations and standard errors are presented.

We selected our top two regions, the *RANKL* and *WNT16* loci, with SNPs with P<1×10^−5^ and carried out analyses conditional on the most associated SNPs in each region: rs9525638 and rs2707466, respectively. When conditioning on the most significant SNP in the *WNT16* region (rs2707466) an additional suggestive signal (rs12706314 in *C7orf58*, P condition = 7.3×10^−5^) appeared, but did not achieve genome-wide significance. Using a similar conditional analysis (with rs9525638) for the *RANKL* locus, no additional SNPs with an independent signal appeared.

### Cortical Thickness Replication Study

**Figure 2:**
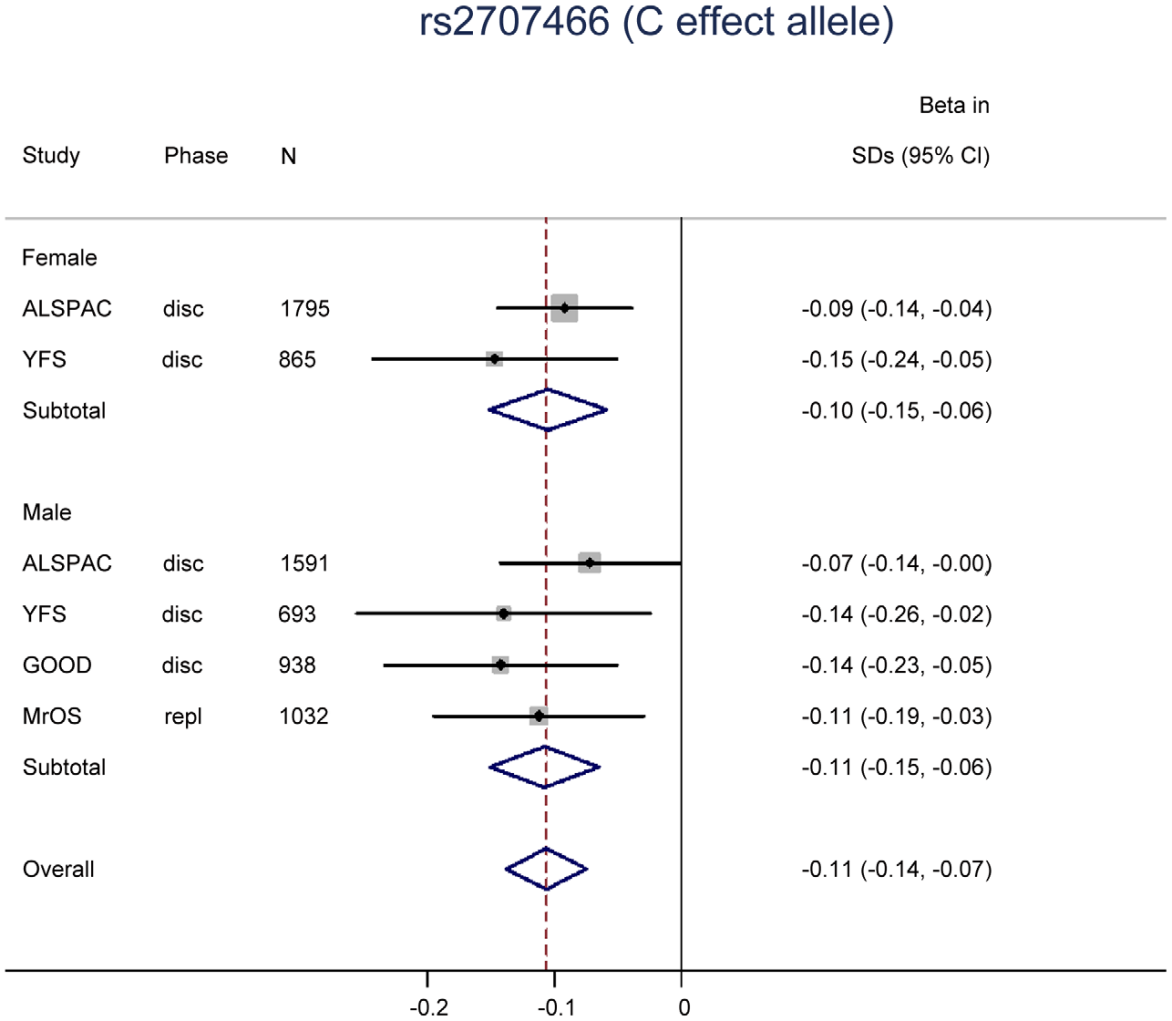
The genome-wide meta-analysis with cortical thickness according to sex.

Two SNPs (rs9525638, rs2707466) were selected for replication in the MrOS Sweden cohort. In the replication stage, SNP rs2707466 at the *WNT16* locus was significantly associated with tibial cortical thickness (−0.11 SD per C allele, P = 0.008), whilst no strong evidence of association was seen for rs9525638 near the *RANKL* locus although the estimated effect was in the same direction as in the discovery meta-analysis (Table 1). Thus, rs2707466 was the only SNP that was significantly associated with cortical thickness in both the discovery and replication cohorts (combined −0.11 SD per C allele, P = 1.5×10^−10^). Therefore, further analysis of associations with bone traits was constrained to rs2707466 at the *WNT16* locus. Associations between rs2707466 and cortical thickness were highly similar when performed according to sex (Figure 2). No evidence of a significant impact of age for the association between rs2707466 and cortical thickness was found (ALSPAC (young) vs. GOOD, YFS and MrOS combined (adult and older): −0.09 SD vs. −0.13 SD per C allele, P = 0.135, for heterogeneity between the two groups). In the combined meta-analysis, rs2707466 was not associated with either cortical vBMD or periosteal circumference (Table S2). In the GOOD cohort, rs2707466 was associated with cortical bone thickness also at the radius (−0.12 SD per C allele, P = 0.008).

### GWAS Meta-Analysis of Forearm BMD

**Figure 3:**
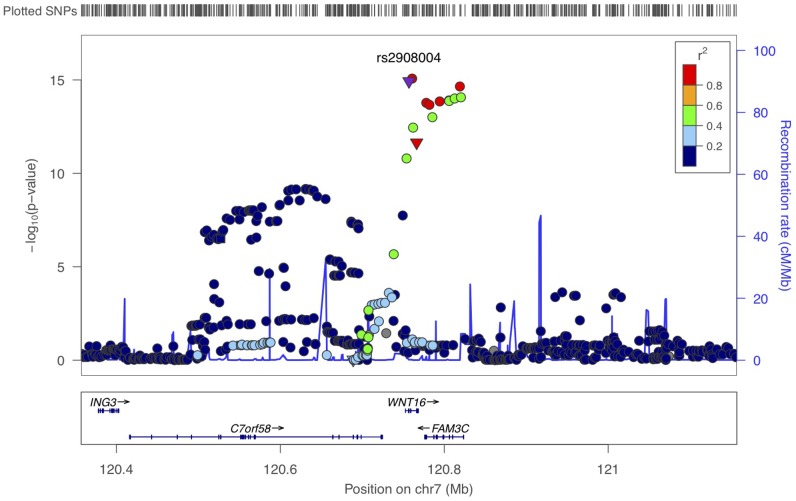
Scatter plots of the observed association of 7q31 locus with forearm BMD. The P values of SNPs (shown as −log10 values in y-axis, from the genome-wide single-marker association analysis using the linear regression model) are plotted against their map position (b36) (x-axis). The color of each SNP spot reflects its r2 with rs2908004. Missense SNPs are plotted as triangles, and other SNPs are plotted as circles.

The information of the five forearm BMD cohorts is presented in Table S3. A quantile-quantile plot of the observed P values showed a clear deviation at the tail of the distribution from the null distribution (Figure S4). The meta-analysis revealed that 54 SNPs within the 7q31 locus had genome-wide significant associations (P<4.6×10^−8^) with forearm BMD (Table S4 and Figure S5). The most significant SNP was at rs2536189 (−0.16 SD per C allele, P = 8.5×10^−16^). Two common amino acid substitutions at *WNT16*, rs2908004 (Gly>Arg) (−0.16 SD per G allele, P = 1.2×10^−15^) and rs2707466 (Thr>Ile as described in cortical thickness study) (−0.14 SD per C allele, P = 2.3×10^−12^) also demonstrated genome-wide significance (Table 2). The highlighted locus at 7q31 locus included genome-wide significant SNPs at the *WNT16* (wingless-type MMTV integration site family, member 16), *FAM3C* (family with sequence similarity 3, member C) and *C7orf58* (chromosome 7 open reading frame 58) genes (Figure 3). To identify the possible secondary signals in this locus, we carried out a conditional analysis. When conditioning on rs2536189, the most significant SNP at *WNT16* for BMD, an additional signal (rs1554634 in *C7orf58*, P = 7.8×10^−8^) was highlighted at a genome-wide suggestive level of association (Figure S6). This SNP is in LD with rs12706314 in *C7orf58* (r^2^ = 0.35 and D' = 0.72 in HapMap CEU), which showed suggestive association with cortical thickness when conditioning on the top signal for cortical thickness.

**Table 2:** Association results of forearm BMD meta-analysis and fracture for selected SNPs.

			Meta Analysis of BMD GWAS					Meta Analysis of Fracture Results					
CHR	SNP	POSITION	EA	NEA	EAF	Beta	P-Value	RA	NEA	OR (95% CI)	P-Value	I2	Gene Annotation
7	rs7776725	120820357	T	C	0.74	−0.17	8.54E−15	T	C	1.33 (1.20–1.46)	7.27E−09	11	FAM3C
7	rs2908004	120757005	G	A	0.58	−0.16	1.17E−15	G	A	1.22(1.12–1.33)	4.90E−06	0	WNT16 Missense
7	rs2707466	120766325	C	T	0.59	−0.14	2.25E−12	C	T	1.22 (1.11–1.33)	7.19E−06	0	WNT16 Missense
7	rs10274324	120686577	T	G	0.94	−0.21	3.82E−08	T	G	1.13 (0.92–1.35)	1.50E−01	0	C7orf58

EA: effect allele; NEA: non-effect allele; EAF: effect allele frequency; RA: risk allele.

See Table S4 for a list of all genome-wide significant SNPs.

### Forearm Fracture Association Study

**Figure 4:**
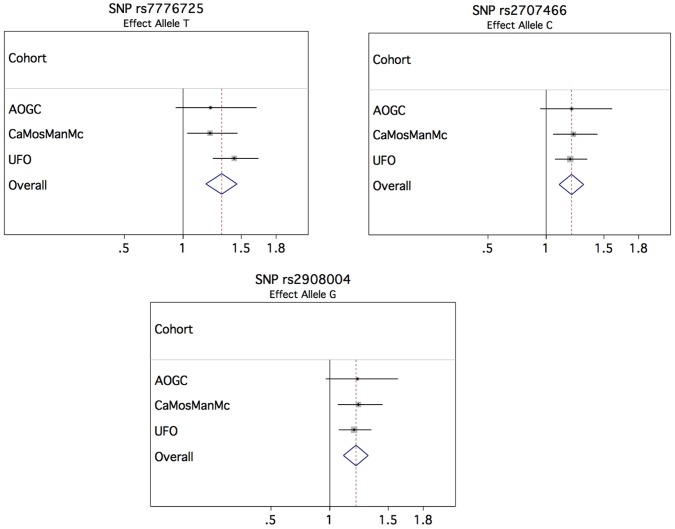
Forest plots of association of top SNPs for forearm fracture.

In order to investigate whether the variants showing association with forearm BMD also have an effect on the risk of forearm fracture, we selected 4 genome-wide significant SNPs from the BMD analysis for de novo genotyping in samples with forearm fracture and their controls (Table S5), including the two missense SNPs in *WNT16* (rs2707466 and rs2908004), one from *FAM3C* (rs7776725) and one from *C7orf58* (rs10274324). In the meta-analysis for osteoporotic fracture, comprising 2,023 forearm fracture cases and 3,740 controls, from 3 cohorts, we identified the rs7776725 SNP in *FAM3C* as being genome-wide significant for forearm fracture, with each C allele increasing the odds of fracture by 1.33 (95% confidence interval [CI]: 1.20–1.46, P-value = 7.3×10^−9^) (Table 2 and Figure 4). The two missense SNPs in *WNT16* also demonstrated strong associations with risk of fracture (rs2908004, risk allele G, OR = 1.22 [95% CI: 1.12–1.33], P-value = 4.9×10^−6^ and rs2707466, risk allele C, OR = 1.22 [95% CI: 1.11–1.33], P-value 7.2×10^−6^). SNP rs10274324 from *C7orf58* was not associated with fracture in this study (P = 0.15). These results were consistent across the three cohorts (Table S6).

### Mouse Gene Deletion Studies

**Figure 5:**
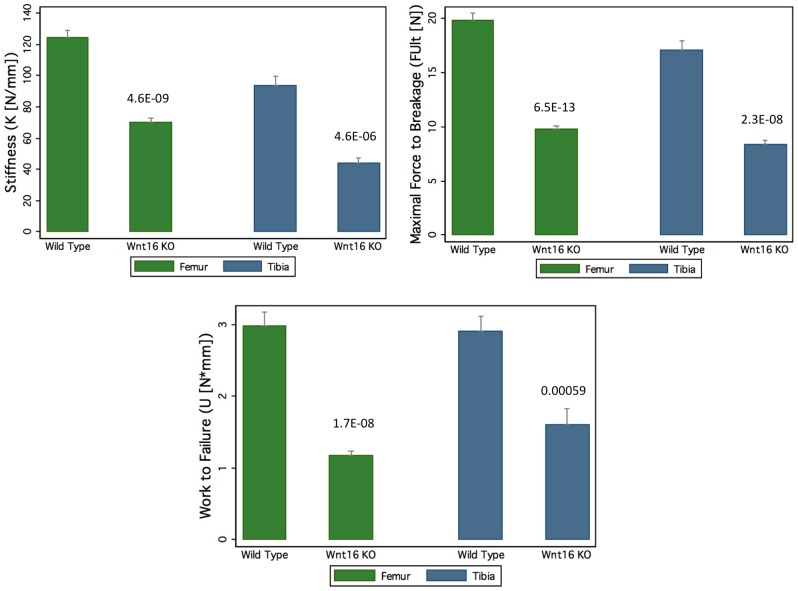
Decrease of bone strength of *Wnt16* knockout mice at femur and tibia. In Femur group, the sample size are 23 wide type (WT) mice and 13 knock out (KO) mice, and in Tibia group, the sample size are 12 WT mice and 9 KO mice. The P values for each group are shown in the figure.

Mice with a gene deletion of *Wnt16* (*Wnt16^−/−^*) appeared healthy with no discernible morphological or growth defects, and had normal body weight and femur length at 24 weeks. In microCT analyses of the femoral diaphysis, male *Wnt16^−/−^* mice had a trend suggestive of reduced cortical thickness (−7%, P = 0.14), and reduced cortical bone polar moment of inertia (−16%, P<0.001) (Table 3); female *Wnt16^−/−^* mice had substantially reduced cortical cross sectional area (−36%, P<0.001) and cortical thickness (−27%, P<0.001) and calculated bone strength (polar moment of inertia, −55%) (Table 3). Trabecular bone volume fraction (bone volume/total volume), as measured by microCT of 5^th^ lumbar vertebrae (LV5), was similar in wild-type and *Wnt16^−/−^* mice (Table 3). In three-point bending tests, measures of bone strength (stiffness, maximal force to breakage and work to failure) were decreased between 43–61% (6.5×10^−13^<P<5.9×10^−4^) in *Wnt16* ^−/−^ female mice at both femur and tibia (Figure 5). However, microCT parameters of the femoral shaft and LV5 in male wild type and *Fam3c^−/−^* mice did not reveal any consistent differences across the three targeting strategies (Table S7).

**Table 3:** Body weight, femoral length, and MicroCT data in *Wnt16^−/−^* mice, males (WT = 9; *Wnt16^−/−^* = 11) and females (WT = 24, *Wnt16^−/−^* = 16).

Parameter	Male WT Mice	Male *Wnt16^−/−^* Mice	Statistics	Female WT Mice	Female *Wnt16^−/−^* Mice	Statistics
Body Weight (grams)	35.4±1.8	37.6±1.3	Δ = ↑10%, P = 0.35	27.3±0.8	25.9±1.3	Δ = ↓5%, P = 0.34
Femur Length (mm)	16.3±0.2	16.2±0.2	Δ = ↑1%, P = 0.58	16.2±0.1	16.1±0.1	Δ = 0%, P = 0.86
LV5 BV/TV (%)	20.0±1.7	22.9±1.1	Δ = ↑15%, P = 0.15	15.0±1.2	16.0±1.1	Δ = ↑7%, P = 0.54
Femur Shaft Total Area (mm^2^)	1.83±0.09	1.60±0.08	Δ = ↓13%, P = 0.07	1.57±0.04	1.07±0.03	Δ = ↓32%, P<0.001
Femur Shaft Bone Area (mm^2^)	0.99±0.04	0.88±0.04	Δ = ↓11%, P = 0.08	0.88±0.02	0.56±0.01	Δ = ↓36%, P<0.001
Femur Shaft Marrow Area (mm2)	0.84±0.06	0.72±0.06	Δ = ↓15%, P = 0.15	0.69±0.03	0.51±0.02	Δ = ↓24%, P<0.001
Femur Shaft Cortical Thickness (µm)	251±9	233±8	Δ = ↓7%, P = 0.14	246±4	186±4	Δ = ↓27%, P<0.001
Femur Shaft Polar MOI (mm^4^)	0.507±0.047	0.428±0.49	Δ = ↓16%, P<0.001	0.376±0.016	0.168±0.008	Δ = ↓55%, P<0.001

## Discussion

Forearm fractures are a common and costly condition. In two separate GWASs for forearm BMD and cortical bone thickness we have identified variants that are genome-wide significant for these traits and, importantly, for forearm fracture at the 7q31 locus. Further, we have provided functional data from mice demonstrating that *Wnt16* ^−/−^ mice have reduced cortical bone thickness and bone strength. These results are among the first to demonstrate a genome-wide significant locus for osteoporotic fracture suggesting that this locus is an important genomic determinant of cortical bone thickness and forearm BMD and fracture as well.

*WNT16* is a member of the wingless-type MMTV integration site family, which has been reported to mediate signaling via canonical or non-canonical *Wnt* pathways. The canonical Wnt pathway has been shown to regulate bone mass. Specifically, loss of function mutations in the Wnt-co receptor *LRP5*, as seen in osteoporosis pseudoglioma syndrome, result in a dramatic loss in bone mass [26], while gain of function mutations give rise to extremely high BMD (5 SD above normal) [27]. *Wnt16* has been proposed to signal via the non-canonical pathway [28], regulating haematopoetic stem cell specification in zebra fish, but whether this signaling system involves regulation of osteoblasts, which are of mesenchymal origin, is unclear. Little is known of the role of *WNT16* in skeletal development and function, but *Wnt16* has previously been implicated in synovial joint development in mice [29]. Several genes involved in the Wnt pathway have been previously identified to be associated with BMD by GWAS. These include known loci *CTNNB* [10], *SOST* [30], *LRP4* [9], [10], *LRP5* [8], [10], *FOXC2* [10], *GPR177* [10], and *MEF2C* [10]. Our study adds *WNT16* to this list of bone-influencing Wnt factors. In addition to our GWA meta-analyses results for cortical bone thickness and forearm BMD, we present a functional study demonstrating that *Wnt16* ^−/−^ mice have a substantial decrease in cortical bone thickness (27%) and bone strength (43–61%), but not bone length. Further, Medina et al (accompanying submission) provide data indicating that variation in *WNT16* also influences BMD in children, suggesting that *WNT16* may influence peak bone mass. Importantly, the clinical relevance of these findings at *WNT16* is supported by our observation that *WNT16* influences clinical fractures in humans and bone strength in mice.

In an experimental study using forearms from cadavers, cortical bone thickness was highly correlated (r = 0.93) to the 3-point bending failure load and could improve the prediction of this strength measure, in combination with bone mineral content derived from DXA [31]. Among individuals suffering a fracture at the radius, cortical bone thickness at the same site was 33% lower than in controls, which was the largest difference observed for the cortical bone traits [32]. The present study constitutes the first GWA study of cortical bone thickness, a trait with crucial importance for bone strength. The SNP rs2707466, which causes a missense amino acid substitution (Thr>Ile) at the *WNT16* locus, was consistently associated with cortical thickness in a meta-analysis of three large discovery cohorts and in a replication cohort (combined P = 1.5×10^−10^). In the GOOD cohort, the rs2707466 was also associated with cortical bone thickness at the radius, with an effect size similar to what was seen for the tibia, indicating that the *WNT16* locus affects cortical bone thickness at both the forearm and leg. Interestingly, the most significant 14 of the 54 genome-wide significant SNPs in the forearm BMD GWAS, were all in the *WNT16* or *FAM3C* genes, and showed high correlation with the two *WNT16* missense SNPs: rs2908004 and rs2707466 (0.58<r2<1, in HapMap CEU data). This finding might suggest that the association signals are driven by these coding SNPs in *WNT16*. These two missense variants were also strongly associated with risk of fracture in our study, but did not achieve genome-wide significance. All together, our results implicate the *WNT16* locus as important for fracture risk, which would likely be mediated via an effect on cortical bone, particularly the thickness of the cortical shell.

*FAM3C*, which is predicted to be expressed in osteoblasts and encodes a newly identified cytokine necessary for epithelial to mesenchymal transition and retinal laminar formation in vertebrates [33]. We identified the SNP rs7776725 within the first intron of *FAM3C* to be genome-wide significant for forearm BMD (P = 8.5×10^−15^) and forearm fracture (P = 8.6×10^−9^). Since the fracture cohorts do not have available BMD data, except for the AOGC cohort, which comprised only 7% of the fracture case population, no meaningful conclusions could be drawn for the independence of the association between fracture and forearm BMD. While candidate gene studies have previously described relationships between genetic variants and fracture [34], [35], [36], we are aware of only one other variant that has been demonstrated to be genome-wide significant for any type of osteoporotic fracture, arising from the *ALDH7A1* gene [12]. Interestingly, SNP rs7776725 in *FAM3C* was previously reported to be associated with speed of sound (SOS) as analyzed by quantitative ultrasound at the radius (P = 1.0×10^−11^) in an un-replicated GWAS carried out in Asian populations [13]. This SNP was also associated with BMD in a Caucasian population [37]. The high-throughput DEXA and microCT screen which initially identified reduced cortical bone thickness and bone strength in *Wnt16* knockout mice failed to observe any skeletal phenotype changes in three independent knockouts of mouse *Fam3c*. Since the sample size of *Fam3c^−/−^* mice was small (N = 18), the possibility of a false negative result cannot be excluded. All together, our functional studies indicate that *Wnt16* rather than *Fam3c* is responsible for the observed genetic signal arising from this locus. However, we provide no data as to whether or not gain of functions variants in *Fam3c* could have effects on the studied bone traits and fracture risk.

*C7orf58* (*FLJ21986*), which codes for a hypothetical protein, has recently been identified to be associated with blood pressure in a study on Nigerians [38]. As an open reading frame, *C7orf58* has no known function. In the forearm GWAS, the other 40 out of the 54 genome wide significant SNPs are from *C7orf58*, and show low LD with rs2908004 and rs2707466 (r2<0.2, in HapMap CEU data), when conditioning for the top SNP (rs2536189) in *WNT16*, resulted in an additional signal (rs1554634) in *C7orf58*. Similarly, conditioning for rs2707466 at the *WNT16* locus, in the GWAS for cortical bone thickness, resulted in an additional, suggestive signal (rs12706314, which was in LD with rs1554634) located in *C7orf58*. Furthermore, Medina et al (accompanying submission) demonstrate in a conditional analysis that a separate signal, other than the signal derived from *WNT16*,, located in *C7orf58* was associated with total body BMD. Thus, these studies reveal an independent genetic signal for several bone traits, arising from *C7orf58*, indicating a possible functional role of this protein. Even though our functional studies imply that *Wnt16* determines the bone effects of the 7q31 locus, further studies are necessary to elucidate the role of *C7orf58*.

In summary, we provide the first evidence of association of common variants across the genome with cortical bone thickness, forearm BMD and forearm fracture. We also provide functional data implicating *WNT16* at this locus. Importantly, our findings report one of two genome-wide significant variants for osteoporotic fracture. These results suggest a critical role of Wnt signaling pathway on cortical bone thickness and bone strength determination as well as fracture susceptibility.

## Materials and Methods

### Ethics Statement

All study participants provided informed written consent. Approval by local institutional review boards was obtained in all studies.

### Study Samples of Bone Cortical Thickness GWAS

#### GOOD cohort

The Gothenburg Osteoporosis and Obesity Determinants (GOOD) study was initiated to determine both environmental and genetic factors involved in the regulation of bone and fat mass [39] [40]. Young men were randomly identified in the greater Gothenburg area in Sweden using national population registers, contacted by telephone, and invited to participate. Enrolled subjects were between 18 and 20 years of age. There were no other exclusion criteria, and 49% of the study candidates agreed to participate (n = 1068). Genotypes from 938 individuals passed the sample quality control criteria (Table S8). We carried out imputation to HapMap2 using Mach 1.0, Markov Chain Haplotyping [41], giving a total of 2,608,508 SNPs

#### YFS cohort

The Cardiovascular Risk in Young Finns Study (YFS) is an ongoing multi-centre follow-up of atherosclerosis risk factors in young Finns [42]. The first cross-sectional survey conducted in 1980 comprised a total of 3,596 subjects (83% of those invited) aged 3, 6, 9, 12, 15 and 18 years. The subjects were randomly selected from the national population register from five university cities in Finland (Helsinki, Turku, Tampere, Kuopio and Oulu) and the rural municipalities in their vicinity. In 2008, 1,884 subjects (1,058 women and 826 men) aged 31–46 years participated in pQCT measurements organized in five study centers (Turku, Helsinki, Tampere, Oulu and Kuopio) between February and December 2008. Trained technologists in each center performed the measurements. The same pQCT device was used in all study centers (Stratec XCT 2000R). Pregnant women were excluded from the pQCT measurements. Subjects gave written informed consent. Bone measures are described in detail elsewhere [43]. Both pQCT measurements and genotype information were available for 1558 study subjects. Genotype imputation was performed using MACH 1.0 [41] and HapMap II CEU samples as the reference set. See more detail in Table S8.

#### ALSPAC cohort

The Avon Longitudinal Study of Parents and their Children (ALSPAC) is a geographically based birth cohort study investigating factors influencing the health, growth, and development of children. All pregnant women resident within a defined part of the former county of Avon in South West England with an expected date of delivery between April 1991 and December 1992 were eligible for recruitment, of whom 14,541 were enrolled (http://www.alspac.bris.ac.uk) [44]. Both mothers and children have been extensively followed from the 8^th^ gestational week onwards using a combination of self-reported questionnaires, medical records and physical examinations. Blood samples were taken and DNA extracted as previously described [45]. 3382 study subjects had both pQCT measurements and genotype information. We carried out imputation using MACH 1.0.16, Markov Chain Haplotyping [41], using CEPH individuals from phase 2 of the HapMap project as a reference set. See more detail in Table S8.

### Study Samples of Bone Cortical Thickness Replication Study

#### MrOS Sweden cohort

The Osteoporotic Fractures in Men (MrOS) study is a prospective multicenter study including older men in Sweden (3014), Hong Kong (>2000), and the United States (>6000). In the present study, associations between candidate polymorphisms and skeletal parameters were investigated in the Swedish cohort (Table 1), which consists of three sub-cohorts from three different Swedish cities (n = 1005 in Malmö, n = 1010 in Gothenburg, and n = 999 in Uppsala). Study subjects were randomly identified using national population registers, contacted and asked to participate. To be eligible for the study, the subjects had to be able to walk without assistance, provide self reported data, and sign an informed consent; there were no other exclusion criteria [46]. See more detail in Table S8.

### Study Samples of Forearm BMD GWAS

5,672 samples from five cohorts of European descent participated in this meta-analysis (Tables S3 and S9). BMD at forearm in all cohorts was measured by dual-energy X-ray absorptiometry following standard manufacturer protocols.

#### TwinUK1 and TwinUK23 cohorts

TwinsUK (http://www.twinsuk.ac.uk/) is a population-based registry of British Twins representative of the general British population [8], [36], [47]. Genotyping of the TwinUK1 was done by Illumina HumanHap300, and TwinUK23 by HumanHap610Q. Imputation was performed using the IMPUTE software package version 2 [48] based on HapMap2, release 22.

#### AFOS cohort

The Amish Family Osteoporosis Study (AFOS) study was designed to identify genetic determinants of osteoporosis in the Old Order Amish (OOA) population from Lancaster County, PA USA [49], [50]. Genotyping was done using either the Affymetrix 500K or 6.0 genotyping chip. The Birdseed genotype-calling algorithm was used. Imputation was performed using MACH on the HapMap2, rel 22 data.

#### GOOD cohort

The GOOD study subjects (study inclusion criteria described under the cortical bone thickness GWA study) were contacted and invited to participate in a five-year follow-up exam [51]. Forearm BMD measurements were available in 731 men (Table S3).

#### AOGC cohort

The Anglo-Australasian Osteoporosis Genetics Consortium (AOGC) study collected unrelated individuals with extreme BMD phenotypes as a powerful strategy for gene discovery in quantitative traits [11], [52]. Genotyping was performed using Illumina Infinium II HumHap370CNVQuad chips at the University of Queensland Diamantina Institute, Brisbane, Australia. Subsequent imputation was done based on the HapMap2 release 22 data using MACH program.

### Study Samples of Forearm Fracture Association Study

Four genome-wide significant SNPs for forearm BMD were selected to test the association with forearm fracture in 2,142 cases and 3,697 controls from three cohorts. Forearm fracture was defined as fractures resulting from low trauma (such as a fall from standing height) occurring at the wrist, ulna, radius, forearm, as well as Colles' fractures.

#### UFO cohort

The Umeå Fracture and Osteoporosis (UFO) study is a nested case-cohort, population-based study from Sweden designed to identify the genetic and gene-by-environmental determinants of osteoporotic fracture. This cohort is sampled from a population-based cohort study from Northern Sweden initiated to identify the risk factors for diabetes and cardiovascular disease [53], [54]. In total, 1,068 cases and 1,218 age-matched controls were included from this cohort. All fractures were confirmed by radiographic or surgical report. De-novo genotyping in the UFO study was undertaken at Kbiosciences (England).

#### CaMos and ManMc cohort

The Canadian MultiCentre Osteoporosis study (CaMos) is a population-based prospective study of 9,423 men and women from across Canada, followed for fourteen years for osteoporotic outcomes and risk factors [55]. All fractures in the CaMos study at forearm were confirmed with radiographic or surgical report. The Manitoba-McGill (ManMc) fracture study is a population-based cohort of women undergoing surgical repair of wrist and hip fractures within the Province of Manitoba, Canada [56]. All individuals had no prior history of concomitant disease or use of bone-altering drugs. All fractures occurred at forearm and were confirmed with surgical report. ManMc was designed to complement the CaMos cohort by recruiting individuals suffering osteoporotic fractures in the most heritable age range (prior to age 70) and compare them to controls from the CaMos cohort that had not suffered an osteoporotic fracture after up to fourteen years of follow-up and subjects reaching at least 70 years of age. Weight and height at time of fracture are not collected for these study participants. 800 cases with forearm fracture and 855 controls were included from the combined CaMos/ManMc study. De novo genotyping for the CaMos and ManMc cohorts at the 4 SNPs assessed for fracture was undertaken at Kbiosciences (England).

#### AOGC cohort

This *in silico* association study included 155 forearm fracture cases and 1,672 controls from the AOGC GWAS [11]. In some samples the level of trauma was not known. In these cases, if the fracture occurred after age 60 years, it was considered to be an osteoporotic low trauma fracture, fractures occurring prior to this age with unknown trauma level were conservatively excluded. Fracture cases were identified by self-report.

### Statistical Analysis

#### Genome-wide meta-analysis and replication method for bone cortical thickness study

The ALSPAC (n = 3382), YFS (n = 1558) and GOOD (n = 938) discovery cohorts contributed to the genome-wide meta-analysis. We analyzed only those imputed SNPs which had a minor allele frequency of >0.01 and an r^2^ imputation quality score of >0.3 in all 3 sets (n = 2,401,124). We carried out genome-wide association analyses for cortical thickness using additive linear regression in Mach2QTL for ALSPAC, ProbABEL [57] for YFS and using GRIMP [58] for the GOOD analyses. We included age, sex, height and weight(ln) as covariates. We carried out meta-analyses of the results from the three cohorts using the inverse variance method. Standardized betas and standard errors from each study are combined using a fixed effect model which weights the studies using the inverse variance and applying genomic control to individual studies and the combined results. Genome-wide significance was taken to be p<5×10^−8^. We selected one SNP from each independent region that had a p<5×10^−8^ for replication in the MrOS Sweden cohort. We also repeated the analyses in each of the three discovery cohorts, conditional on these top SNPs, to identify any additional independent associations in the regions. Additive linear regression analyses were carried out for the associations between these SNPs and cortical thickness in SPSS Statistics 17.0 for MrOS Sweden, using age, sex, height and weight(ln) as covariates. The results of all four cohorts were combined using a fixed effects inverse-variance meta-analysis in Stata (version 11.2). Correlations between bone traits in the MrOS cohort were tested and presented as Spearman's rank correlation coefficients (rho).

#### Genome-wide meta-analysis method for forearm BMD study

All cohorts independently conducted the association analysis of SNP allele dosage with standardized BMD residuals, while adjusting for age, age^2^, gender, weight and population substructure where applicable, for centre of recruitment (AOGC), and for family structure in cohorts with family members. The analyses were performed for men and women combined. Details of each study's GWAS are found on Table S9. A meta-analysis of the GWAS results was conducted using the GWAMA software (Genome-Wide Association Meta Analysis) (http://www.well.ox.ac.uk/gwama/) [59], with which meta-analyses are performed for both directly genotyped and imputed SNPs using estimates of the allelic effect size and standard error for BMD, and estimates of the allelic odds ratio and 95% confidence interval for fracture. Poorly imputed SNPs (r2 in MACH<0.3 or proper_info in IMPUTE2<0.40) and SNPs with low MAF (<0.01) were excluded. The summary effect estimates for BMD and fracture risk were computed using fixed-effects inverse variance meta-analysis [60]. Cochran's Q statistic and I^2^ estimates were used to evaluate the heterogeneity. To control for possible inflation of statistics due to population stratification and family relations, genomic control was applied to each study as well as the overall meta-analysis [61]. Statistical significance for genome-wide BMD association study was set at P<5×10^−8^.

#### Fracture association analysis

Four of the 54 genome-wide significant SNPs, in the forearm BMD GWAS, were selected to test the association with fracture. The rationale for picking these SNPs included: 1) Given that the Wnt pathway is central to osteoporosis etiology, two missense SNPs (rs2908004 and rs2707466) from *WNT16* were selected, considering that missense SNPs may have more functional consequence than synonymous or non-coding SNPs, and that these two SNPs can fully tag the top SNP rs2536189 (r2>0.98); 2) The SNP rs7776725, which is in *FAM3C*, was selected because it was previously reported to be associated with speed of sound through bone, as analyzed by quantitative ultrasound at the radius [13] and was in only moderate LD with the rs2908004 (r2 = 0.58); 3) The SNP rs10274324 was selected based on its genome wide significance (−0.21 SD per T allele, P = 3.8×10^−8^ for forearm BMD), LD information (r2 = 0.04 with the top SNP rs2536189) and location (in *C7orf58* gene). Therefore, the SNP selection provided assessment across all three genes at this locus (*WNT16*, *FAM3C* and *C7orf58*). SNPs were assessed for association with fracture risk using logistic regression models adjusted for sex, height and weight. Age was included as an additional covariate where this was not controlled for through the study design. Again, a fixed effect meta-analysis was undertaken assessing the effect of allelic dose on risk of fracture.

### Generation of Knockout Mice

#### *Wnt16^−/−^* mice

Mice with a gene deletion of *Wnt16* were generated using homologous recombination techniques. The first three exons were disrupted, with confirmation by Southern hybridization analyses (Figure S7). F2 hybrid littermates, derived from C57BL/6J and 129 SvEv parental strains, were examined at 24 weeks of age.

#### *Fam3c^−/−^* mice

Three separate knockout strategies were employed to inactive mouse *Fam3c*: 1) gene trap disrupting the intron between the first two exons with confirmation by lack of gene expression by RT-PCR in kidney and spleen (Figure S8), 2) homologous recombination removing the first two coding exons with confirmation by Southern hybridization analysis (Figure S9), 3) homologous recombination involving replacement of the mouse gene by the human gene resulting in loss of function (Figure S10). F2 hybrid littermates, derived from C57BL/6J and 129 SvEv parental strains, were examined at 16 weeks of age. All studies were performed in accordance with institutional and regulatory guidelines for animal care.

#### Imaging

Male and female mice were scanned using a microCT (Scanco μCT40, Switzerland). The fifth lumbar vertebrae (LV5) were scanned with a voxel size of 16 µm. Midshaft femurs were scanned with a voxel size of 20 µm. All scans used a threshold of 240, an X-ray tube voltage of 55 keV, a current of 145 microamperes and an integration time of 200 microseconds. Three-point bending tests were performed using Mach-1TM Micromechanical System A300.100 (Bio Syntech Canada inc., Laval, Quebec, Canada). The extrinsic parameters (ultimate force [Fult], stiffness [K or S], and work to failure [W or U]) were determined from a force-displacement curve. The span of two support points was 7 mm. The de-formation rate was 50 µm/s.

#### Statistical analysis

Two-sided student's t-test was employed to determine statistical significance of the effect of gene inactivation for each gender. Results are shown as mean+SEM.

## Supporting Information

Figure S1 Quantile-quantile plots of the observed P values versus the expected P values for association for GWAS Meta-Analysis of cortical thickness. The scatters in black showed a clear deviation at the tail of the distribution from the null distribution (the red line).
(DOCX)

Figure S2 Manhattan plot for GWAS Meta-Analysis of cortical thickness. Genome-wide P values (−log10 P) of the linear regression analysis plotted against position on each chromosome.
(DOCX)

Figure S3 SNP rs9525638 regional association plot of the discovery genome-wide meta-analysis of cortical thickness. Circles show GWA meta-analysis p-values, with different colors indicating varying linkage disequilibrium with rs9525638 (diamond).
(DOCX)

Figure S4 Quantile-quantile plots of the observed P values versus the expected P values for association of Forearm BMD. The scatters in blue were based on the entire set of SNPs, whereas the scatters in black were obtained after removing WNT16 region SNPs (+/−400KB either side of rs2908004). The black line was the distribution expected if there were no association.
(DOCX)

Figure S5 Manhattan plot for GWAS Meta-Analysis of Forearm BMD. Genome-wide P values (−log10 P) of the linear regression analysis plotted against position on each chromosome.
(DOCX)

Figure S6 Scatter plots of the observed association of 7q31 locus with forearm BMD after condition on the top SNP rs2536189. The P values of SNPs (shown as −log10 values in y-axis, from the genome-wide single-marker association analysis using the linear regression model) are plotted against their map position (b36) (x-axis).
(PDF)

Figure S7 A: Restriction map of the *Wnt16* gene and construction of the neomycin-resistance (neo) vector. *Wnt16* exons are shown as filled boxes. Sequence information (deletion, insertion site, flanking sequence) is provided on the Taconic Farms website (http://www.taconic.com/wmspage.cfm?parm1=16 catalogue number TF3785). B: confirmation by Southern blots.
(DOCX)

Figure S8 A: Retroviral insertion disrupted *Fam3c* gene prior to the exon encoding amino acid 19 in a protein of 227 amino acids. Sequence information (deletion, insertion site, flanking sequence) is provided on the Taconic Farms website (http://www.taconic.com/wmspage.cfm?parm1=16 catalogue number TF3786). B: RT-PCR analysis revealed that the wild-type transcript was absent in the (−/−) mouse analyzed. Larger transcripts were detected at low levels in both tissues of the (−/−) mouse due to the splicing of fragments from the retroviral vector into the target transcript as determined by nucleotide sequence analysis. However, the in-frame stop codon in the retroviral vector sequence was predicted to disrupt translation of this transcript.
(DOCX)

Figure S9 A: homologous recombination removing the first two coding exons of *Fam3c*. Sequence information (deletion, insertion site, flanking sequence) is provided on the Taconic Farms website (http://www.taconic.com/wmspage.cfm?parm1=16 catalogue number TF3787). B: confirmation by Southern hybridization analysis.
(DOCX)

Figure S10 A: homologous recombination involving replacement of the mouse gene by the human gene resulting in loss of function of *Fam3c*. Sequence information (deletion, insertion site, flanking sequence) is provided on the Taconic Farms website (http://www.taconic.com/wmspage.cfm?parm1=16 catalogue number TF3788). B: confirmation by Southern hybridization analysis.
(DOCX)

Table S1 Characteristics of the included cohorts for GWAS meta-analysis of cortical bone thickness.
(DOCX)

Table S2 SNP rs2707466 associations with pQCT derived bone parameters at different ages and meta-analyses results for cortical bone thickness study.
(DOCX)

Table S3 Characteristics of the included cohorts for GWAS meta-analysis of forearm BMD.
(DOCX)

Table S4 54 genome-wide significant SNPs in 7q31 for forearm BMD GWAS meta-analysis.
(XLSX)

Table S5 Characteristics of the included cohorts for fracture study.
(DOCX)

Table S6 Association results for the 3 fracture cohorts.
(DOCX)

Table S7 Micro CT parameters of the femoral shaft and fifth lumbar vertebra (LV5) in male wild type and *Fam3c*−/− mice. Data are presented for each of the three KO strategies and also for the combined cohorts (WT = 2; *Fam3c*−/− = 4 for each of the individual cohorts).
(DOCX)

Table S8 Bone measurement, genotyping, quality control, imputation by study for cortical bone thickness meta-analysis.
(XLSX)

Table S9 Genome-wide genotyping, imputation and genotype-phenotype analysis by study for BMD meta-analysis.
(XLSX)
