# Supplementary figures and images for: WNT16 Influences Bone Mineral Density, Cortical Bone Thickness, Bone Strength, and Osteoporotic Fracture Risk
Source: PLoS Genet. 2012 Jul 5;8(7):e1002745. doi: 10.1371/journal.pgen.1002745 (PMC3390364; doi:10.1371/journal.pgen.1002745)

**Figure S1**

**
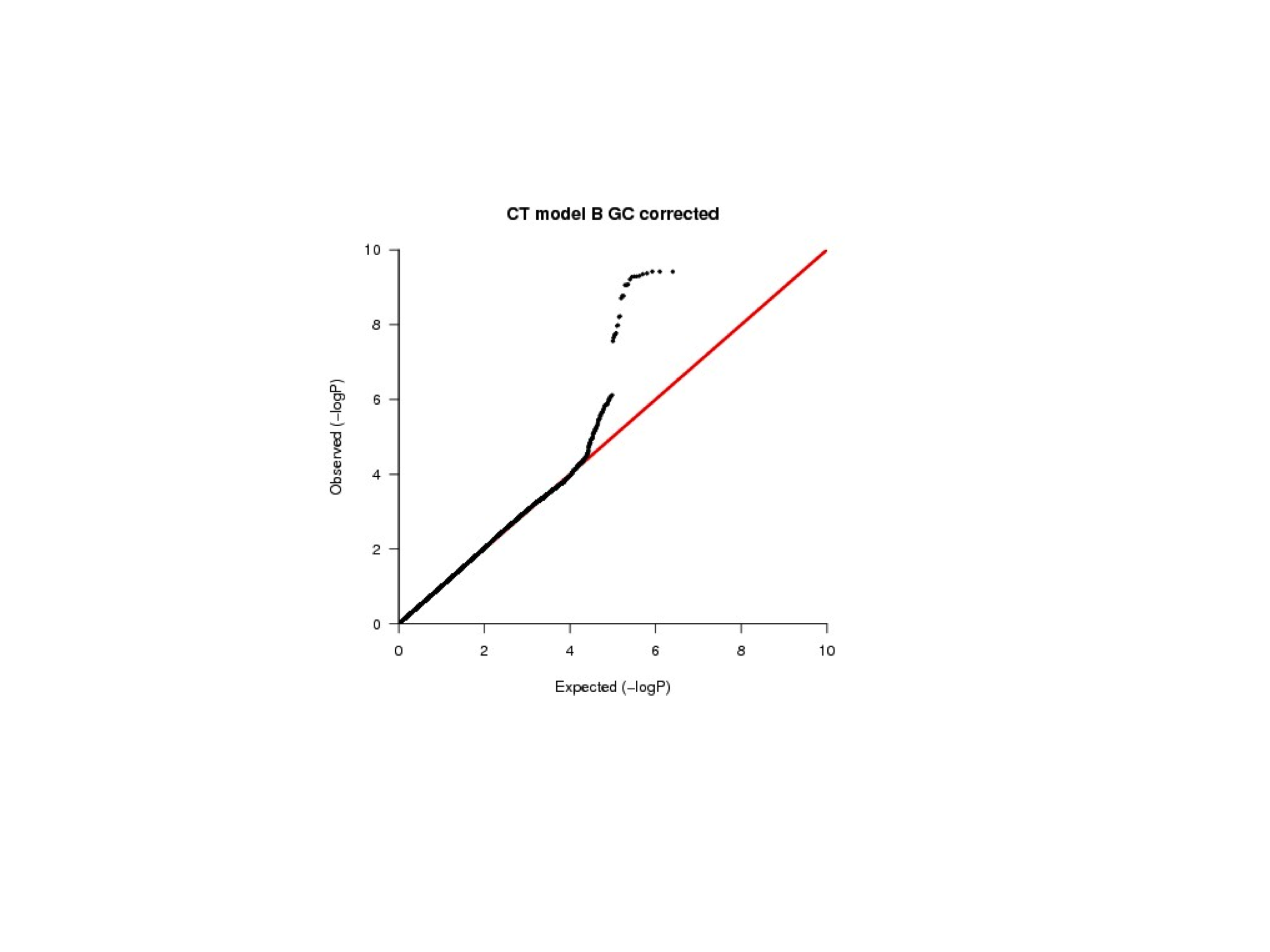
**

Supplement: Figure S1 — Quantile-quantile plots of the observed P values versus the expected P values for association for GWAS Meta-Analysis of cortical thickness. The scatters in black showed a clear deviation at the tail of the distribution from the null distribution (the red line). (DOCX) [file pgen.1002745.s001.docx]

**Figure S2**

**
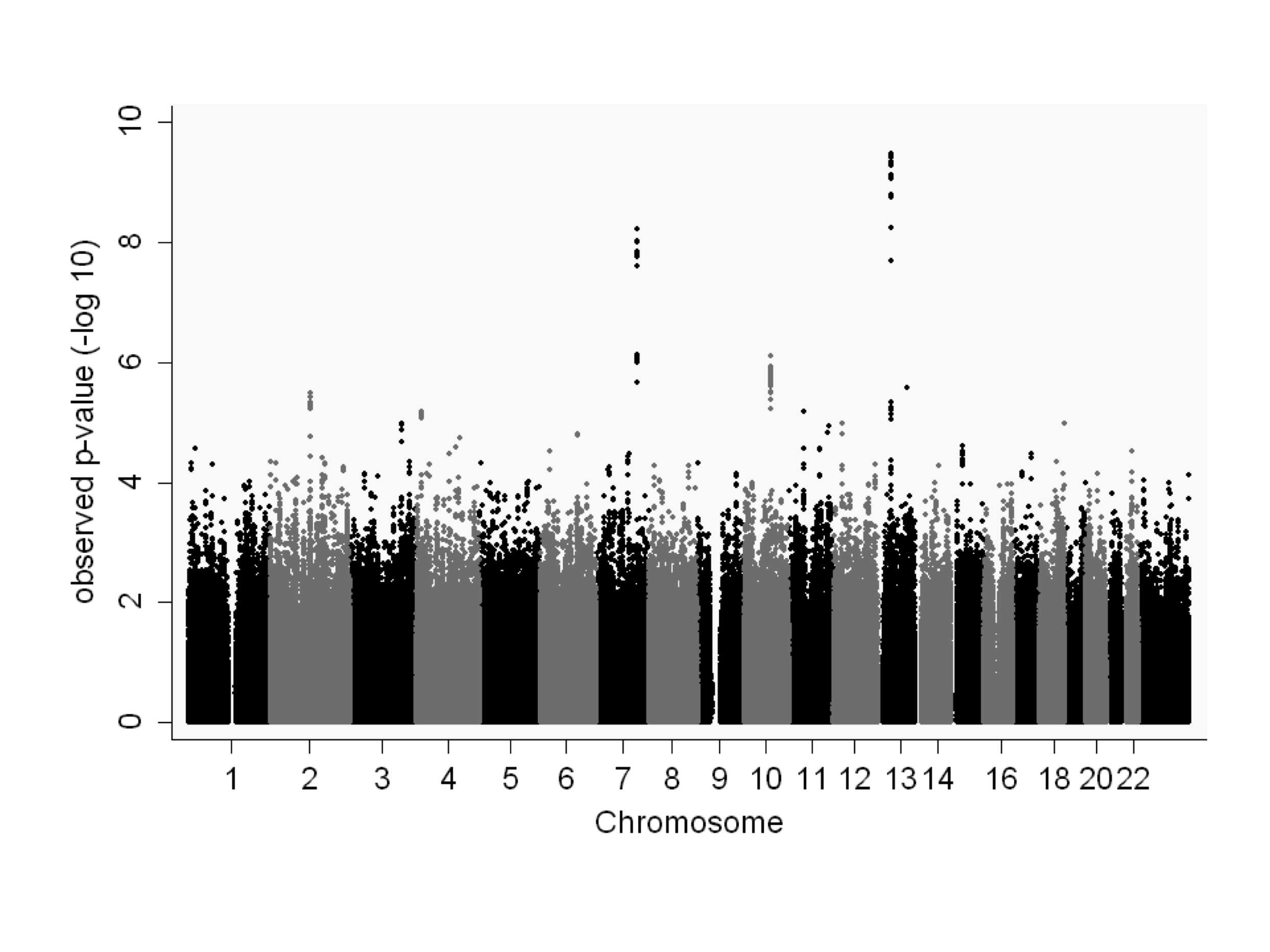
**

Supplement: Figure S2 — Manhattan plot for GWAS Meta-Analysis of cortical thickness. Genome-wide P values (−log10 P) of the linear regression analysis plotted against position on each chromosome. (DOCX) [file pgen.1002745.s002.docx]

**Figure S3**

**
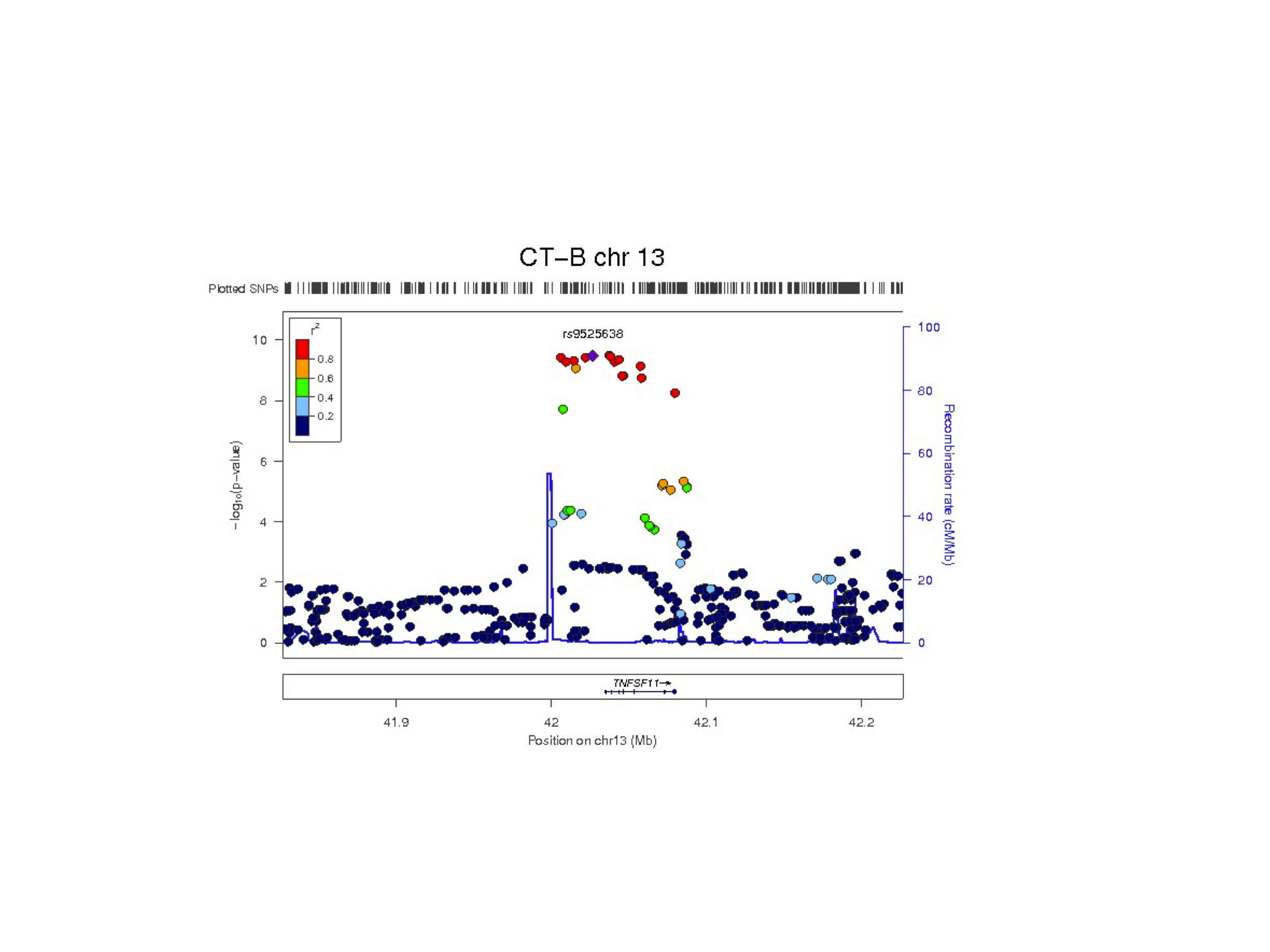
**

Supplement: Figure S3 — SNP rs9525638 regional association plot of the discovery genome-wide meta-analysis of cortical thickness. Circles show GWA meta-analysis p-values, with different colors indicating varying linkage disequilibrium with rs9525638 (diamond). (DOCX) [file pgen.1002745.s003.docx]

**Figure S4**


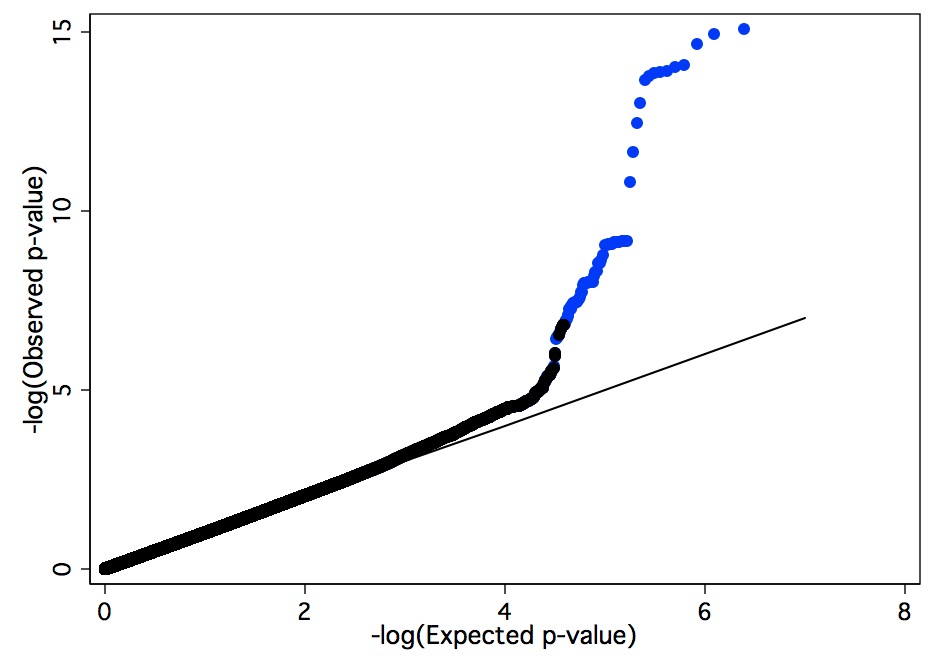

Supplement: Figure S4 — Quantile-quantile plots of the observed P values versus the expected P values for association of Forearm BMD. The scatters in blue were based on the entire set of SNPs, whereas the scatters in black were obtained after removing WNT16 region SNPs (+/−400KB either side of rs2908004). The black line was the distribution expected if there were no association. (DOCX) [file pgen.1002745.s004.docx]

**Figure S5**

**
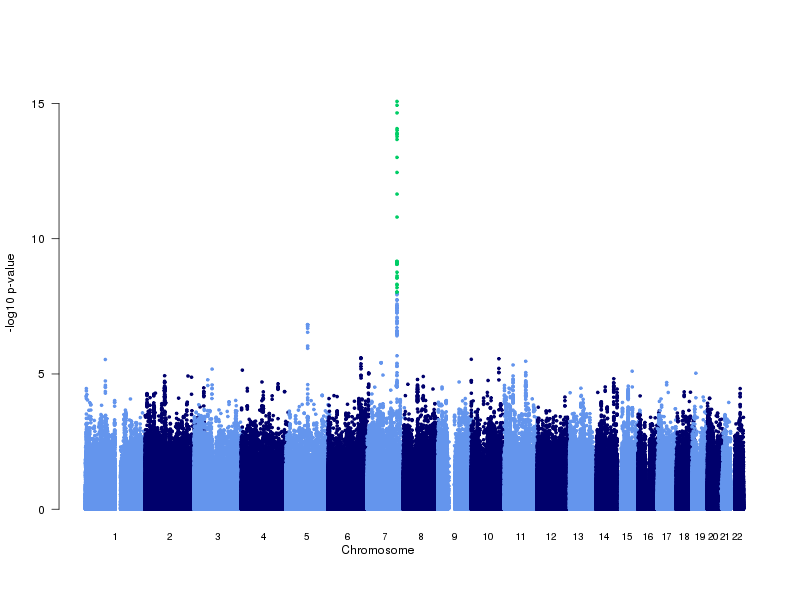
**

Supplement: Figure S5 — Manhattan plot for GWAS Meta-Analysis of Forearm BMD. Genome-wide P values (−log10 P) of the linear regression analysis plotted against position on each chromosome. (DOCX) [file pgen.1002745.s005.docx]

**Figure S7**

**
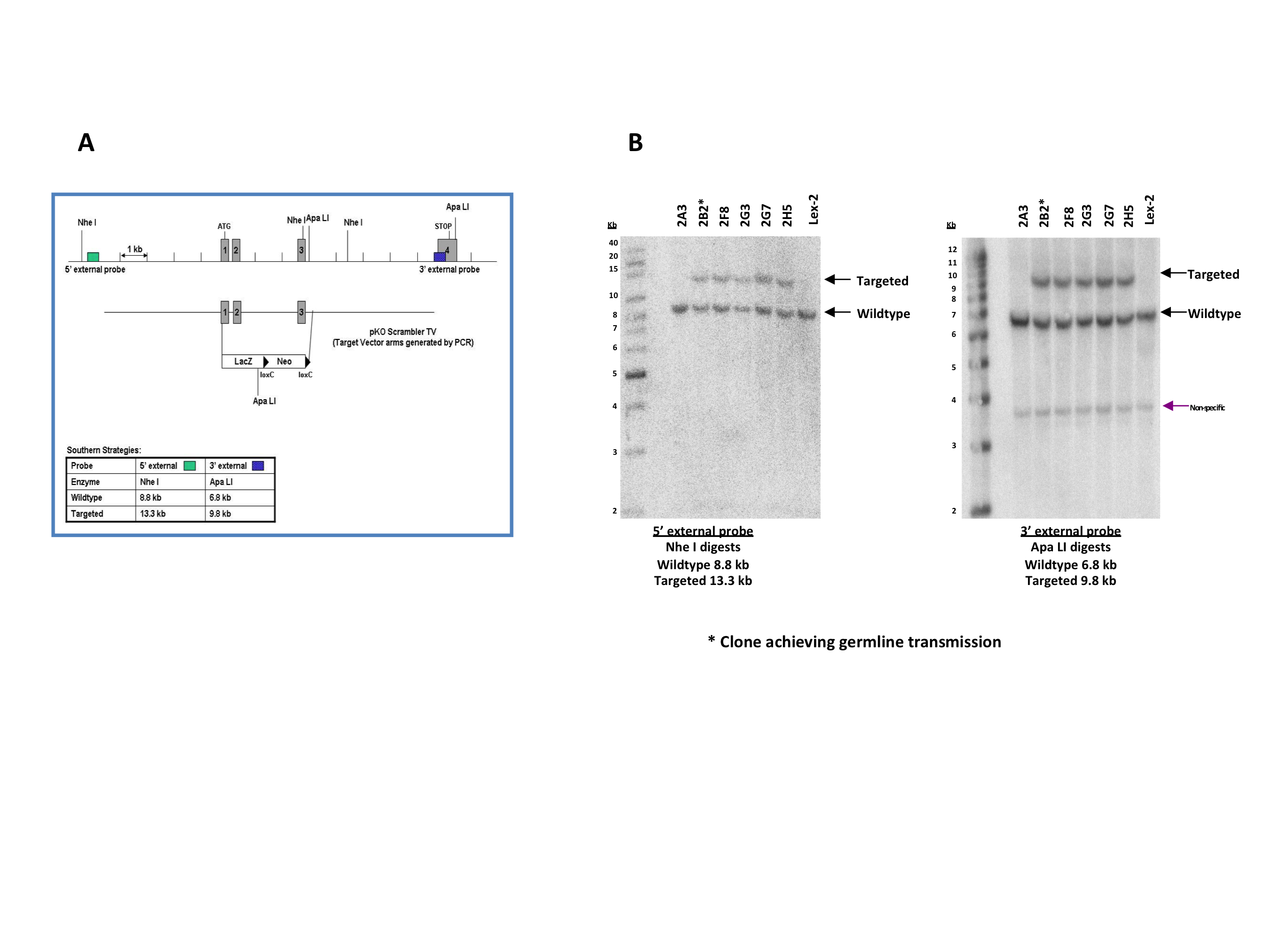
**

Supplement: Figure S7 — A: Restriction map of the Wnt16 gene and construction of the neomycin-resistance (neo) vector. Wnt16 exons are shown as filled boxes. Sequence information (deletion, insertion site, flanking sequence) is provided on the Taconic Farms website (http://www.taconic.com/wmspage.cfm?parm1=16 catalogue number TF3785). B: confirmation by Southern blots. (DOCX) [file pgen.1002745.s007.docx]

**Figure S8**

**
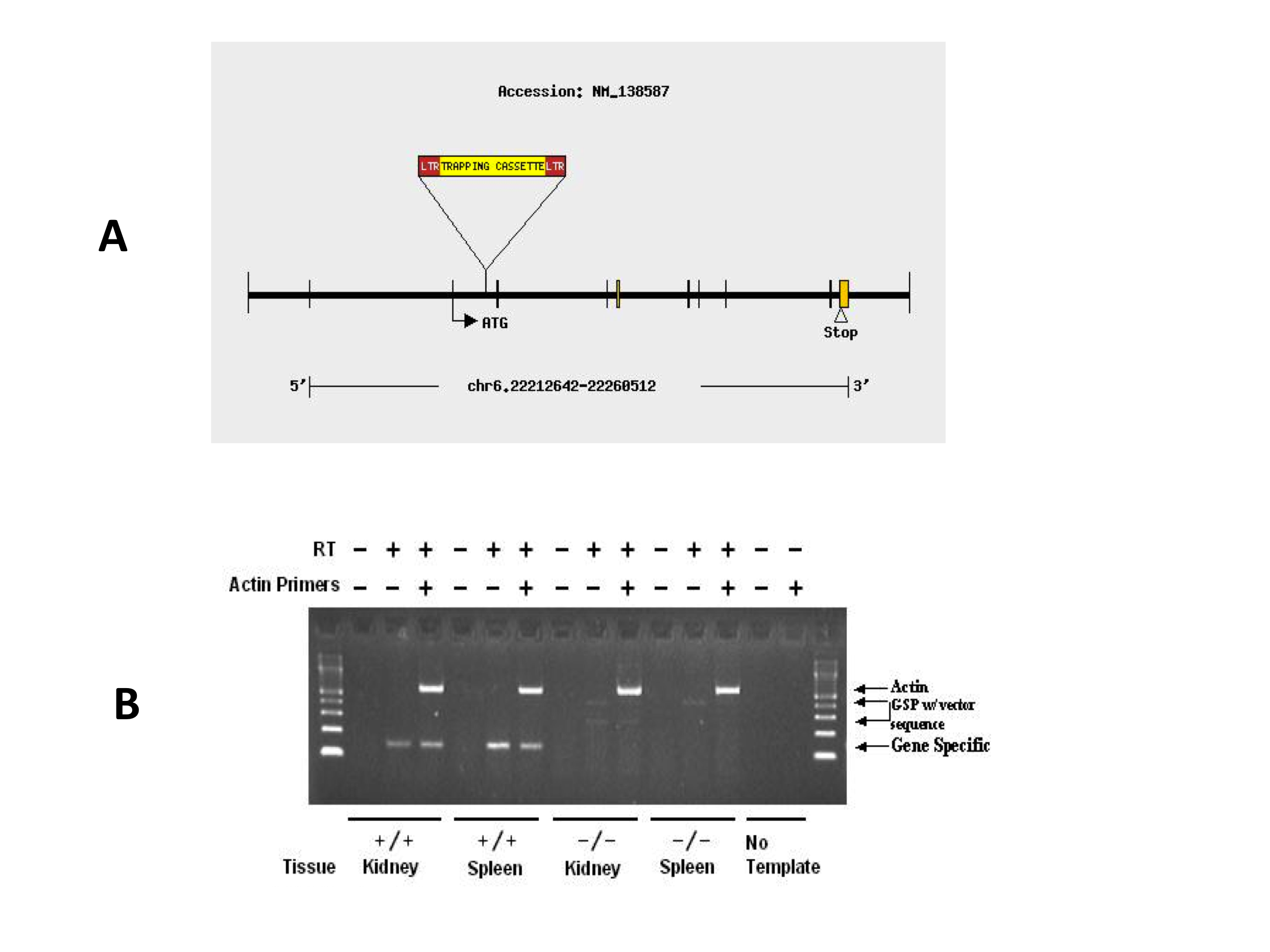
**

Supplement: Figure S8 — A: Retroviral insertion disrupted Fam3c gene prior to the exon encoding amino acid 19 in a protein of 227 amino acids. Sequence information (deletion, insertion site, flanking sequence) is provided on the Taconic Farms website (http://www.taconic.com/wmspage.cfm?parm1=16 catalogue number TF3786). B: RT-PCR analysis revealed that the wild-type transcript was absent in the (−/−) mouse analyzed. Larger transcripts were detected at low levels in both tissues of the (−/−) mouse due to the splicing of fragments from the retroviral vector into the target transcript as determined by nucleotide sequence analysis. However, the in-frame stop codon in the retroviral vector sequence was predicted to disrupt translation of this transcript. (DOCX) [file pgen.1002745.s008.docx]

**Figure S9**

**
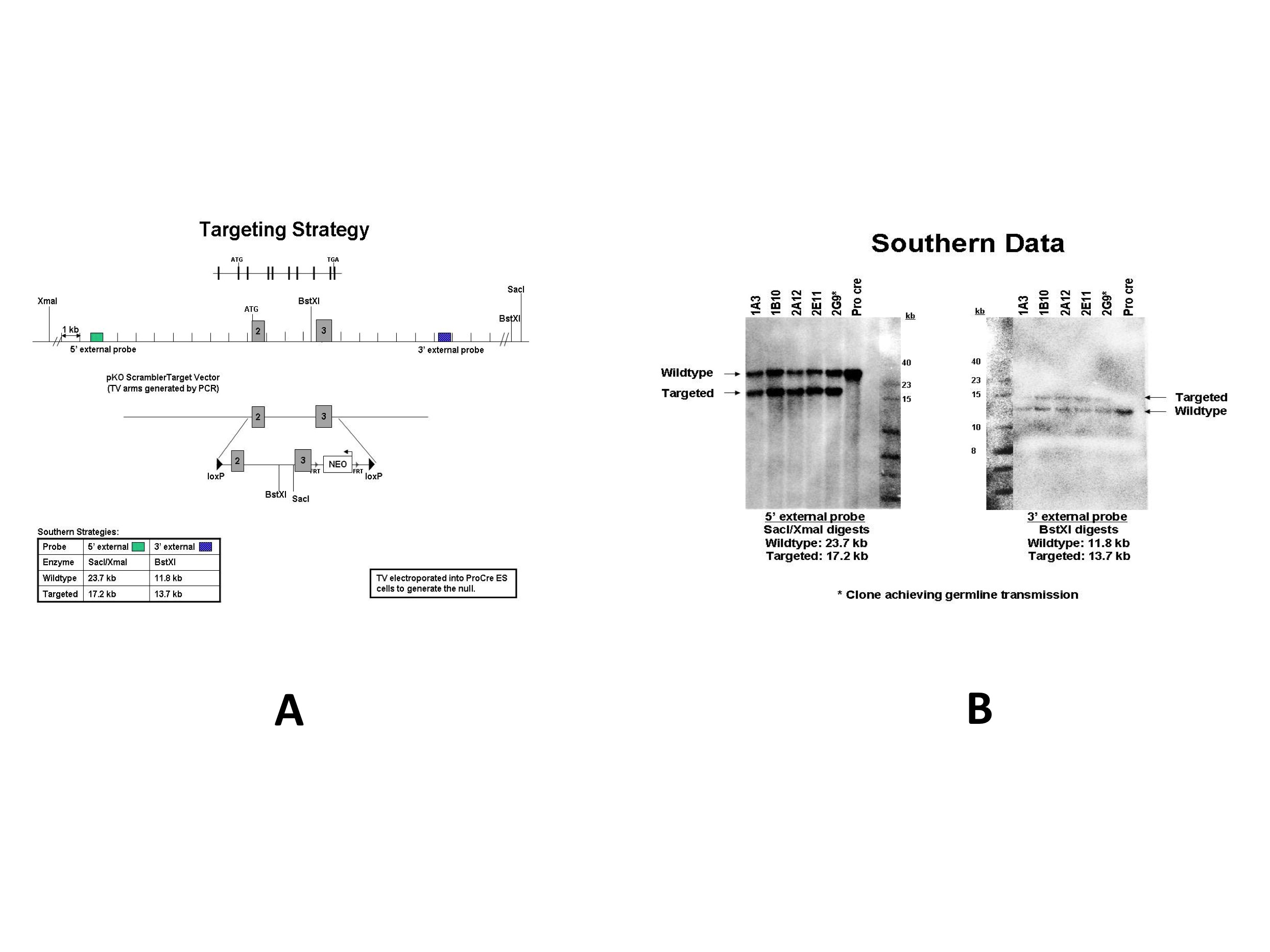
**

Supplement: Figure S9 — A: homologous recombination removing the first two coding exons of Fam3c. Sequence information (deletion, insertion site, flanking sequence) is provided on the Taconic Farms website (http://www.taconic.com/wmspage.cfm?parm1=16 catalogue number TF3787). B: confirmation by Southern hybridization analysis. (DOCX) [file pgen.1002745.s009.docx]

**Figure S10**

**
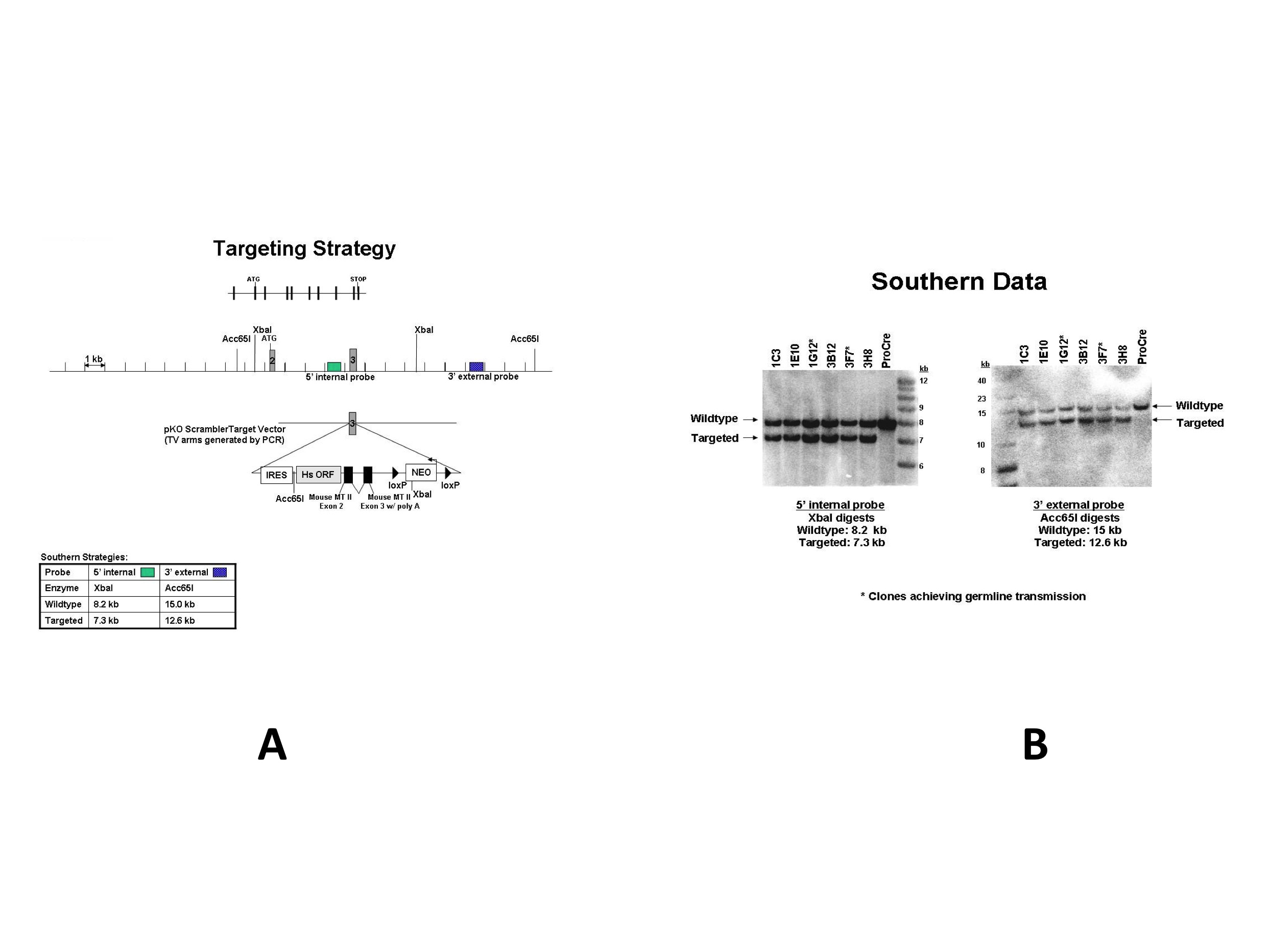
**

Supplement: Figure S10 — A: homologous recombination involving replacement of the mouse gene by the human gene resulting in loss of function of Fam3c. Sequence information (deletion, insertion site, flanking sequence) is provided on the Taconic Farms website (http://www.taconic.com/wmspage.cfm?parm1=16 catalogue number TF3788). B: confirmation by Southern hybridization analysis. (DOCX) [file pgen.1002745.s010.docx]
