# Supplementary material for: WNT16 Influences Bone Mineral Density, Cortical Bone Thickness, Bone Strength, and Osteoporotic Fracture Risk
Source: PLoS Genet. 2012 Jul 5;8(7):e1002745. doi: 10.1371/journal.pgen.1002745 (PMC3390364; doi:10.1371/journal.pgen.1002745)

Supplementary Figure S6

Scatter plots of the observed association of 7q31 locus with forearm BMD after condition on the top SNP rs2536189. The P values of SNPs (shown as –log10 values in y-axis, from the genome-wide single-marker association analysis using the linear regression model) are plotted against their map position (b36) (x-axis).

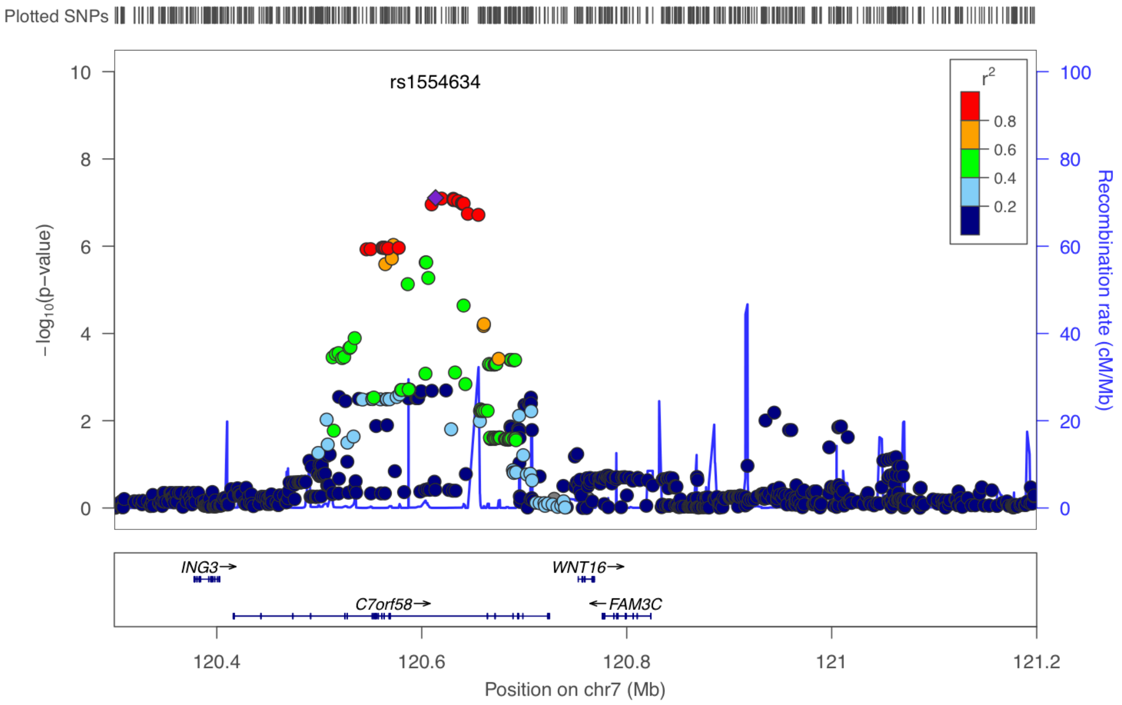

Supplement: Figure S6 — Scatter plots of the observed association of 7q31 locus with forearm BMD after condition on the top SNP rs2536189. The P values of SNPs (shown as −log10 values in y-axis, from the genome-wide single-marker association analysis using the linear regression model) are plotted against their map position (b36) (x-axis). (PDF) [file pgen.1002745.s006.pdf]
