# Supplementary material for: WNT16 Influences Bone Mineral Density, Cortical Bone Thickness, Bone Strength, and Osteoporotic Fracture Risk
Source: PLoS Genet. 2012 Jul 5;8(7):e1002745. doi: 10.1371/journal.pgen.1002745 (PMC3390364; doi:10.1371/journal.pgen.1002745)
Supplement: Table S1 — Characteristics of the included cohorts for GWAS meta-analysis of cortical bone thickness. (DOCX) [file pgen.1002745.s011.docx]

| **Table S1.** Characteristics of the included cohorts for GWAS meta-analysis of cortical thickness. | | | | | | | | | |
| --- | --- | --- | --- | --- | --- | --- | --- | --- | --- |
|  |  |  |  | **Discovery** | |  |  | **Replication** | |
|  |  | ALSPAC |  | YFS |  | GOOD |  | MrOs |  |
|  |  | N=3382 |  | N=1558 |  | N=938 |  | N=1032 | |
|  |  |  |  |  |  |  |  |  |  |
| Age, years |  | 15.5 | (0.3) | 38.0 | (5.0) | 18.9 | (0.6) | 78.7 | (3.0) |
| Men, no. (%) |  | 1587 | (47) | 693 | (44.5) | 938 | (100) | 1032 | (100) |
| Height, cm |  | 169.3 | (8.3) | 172.1 | (9.0) | 181.7 | (6.6) | 173.9 | (6.4) |
| Weight, kg |  | 61.2 | (11.3) | 77.0 | (16.4) | 73.9 | (11.6) | 79.2 | (11.2) |
| Position of cortical section from distal end of tibia |  | 50% |  | 30% |  | 25% |  | 38% |  |
| Cortical | vBMD, mg/cm^3^ | 1101 | (38.3) | 1159 | (23.7) | 1156 | (19.9) | 1128 | (40.8) |
|  | Th, mm | 5.4 | (0.7) | 5.3 | (0.7) | 4.4 | (0.5) | 5.2 | (0.75) |
|  | PC, mm | 72.6 | (6.1) | 69.3 | (6.0) | 75.0 | (4.9) | 79.6 | (5.9) |
| Values presented are means and (SD) |  |  |  |  |  |  |  |  |  |
