# Supplementary material for: WNT16 Influences Bone Mineral Density, Cortical Bone Thickness, Bone Strength, and Osteoporotic Fracture Risk
Source: PLoS Genet. 2012 Jul 5;8(7):e1002745. doi: 10.1371/journal.pgen.1002745 (PMC3390364; doi:10.1371/journal.pgen.1002745)
Supplement: Table S2 — SNP rs2707466 associations with pQCT derived bone parameters at different ages and meta-analyses results for cortical bone thickness study. (DOCX) [file pgen.1002745.s012.docx]

| **Table S2.** SNP rs2707466 associations with pQCT derived bone parameters at different ages and meta-analyses results | | | | | | | | | | | | | | | | | | | | | | | | | | |
| --- | --- | --- | --- | --- | --- | --- | --- | --- | --- | --- | --- | --- | --- | --- | --- | --- | --- | --- | --- | --- | --- | --- | --- | --- | --- | --- |
|  |  |  | **Discovery** | | | |  |  |  |  |  |  |  | **Meta-** **analysis** | | |  | **Replication** | | | **All cohorts** | | | | |  |
|  |  |  | **Alspac discovery** | | |  | **GOOD discovery** | | | **YFS discovery** | | |  |  |  |  |  | **MrOs Sweden** | | | **combined** | | | | |  |
|  |  |  |  |  |  |  | **19-years** | |  |  |  |  |  |  |  |  |  | **70-years** | |  |  |  | |  | |  |
| **Cortical** |  | **Effect** |  | **Beta** |  |  |  | **Beta** |  |  | **Beta** | |  |  |  | **Beta** |  |  | **Beta** |  |  | **Beta** | |  | |  |
| **bone parameters** |  | **allele** | **N** | **(se)** | **p** |  | **N** | **(se)** | **p** | **N** | **(se)** | **p** |  | **N** |  | **(se)** | **p** | **N** | **(se)** | **p** | **N** | **(se)** | | **p** | |  |
| Volumetric BMD | | C | 3382 | 0.02 | 0.41 |  | 938 | -0.05 | 0.44 | 1558 | -0.10 | 6.4E-03 |  | 5878 |  | -0.01 | 0.48 | 1032 | -0.07 | 0.08 | 6910 | -0.02 | | 0.19 | |  |
| (mg/cm^3^) |  |  |  | (0.02) | |  |  | (0.05) |  |  | (0.04) | |  |  |  | (0.02) |  |  | (0.04) | |  | | (0.01) | |  |  |
| Periosteal |  | C | 3382 | 0.01 | 0.43 |  | 938 | 0.03 | 0.41 | 1558 | 0.04 | 0.28 |  | 5878 |  | 0.02 | 0.18 | 1032 | 0.03 | 0.46 | 6910 | 0.02 | | 0.12 | |  |
| Circumference (mm) | |  |  | (0.02) | |  |  | (0.04) |  |  | (0.04) | |  |  |  | (0.01) |  |  | (0.04) | |  | | (0.01) | |  |  |
| Thickness |  | C | 3382 | -0.08 | 1.6E-04 | | 938 | -0.14 | 2.2E-03 | 1558 | -0.14 | 1.7E-04 |  | 5878 |  | -0.10 | 5.9E-09 | 1032 | -0.11 | 8.0E-03 | 6910 | -0.11 | | 1.5E-10 | |  |
| (mm) |  |  |  | (0.02) | |  |  | (0.05) |  |  | (0.04) | |  |  |  | (0.02) |  |  | (0.04) | |  | | (0.02) | |  |  |

Models adjusted for sex (ALSPAC and YFS), age, height, weight (ln). Betas in standard deviations and standard errors are presented
