# Supplementary material for: WNT16 Influences Bone Mineral Density, Cortical Bone Thickness, Bone Strength, and Osteoporotic Fracture Risk
Source: PLoS Genet. 2012 Jul 5;8(7):e1002745. doi: 10.1371/journal.pgen.1002745 (PMC3390364; doi:10.1371/journal.pgen.1002745)
Supplement: Table S3 — Characteristics of the included cohorts for GWAS meta-analysis of forearm BMD. (DOCX) [file pgen.1002745.s013.docx]

| **Table S3.** Characteristics of the included cohorts for GWAS meta-analysis of forearm BMD. | | | | | | | |
| --- | --- | --- | --- | --- | --- | --- | --- |
| Study | Sample Size | Sample Size | Age | Height (cm) | Weight (kg) | Genotyping platform | BMD measurement |
|  | Total | male/female | mean/sd | mean/sd | mean/sd |  |  |
| AFOS | 829 | 403/426 | 52.3/15.1 | 165/8.6 | 75.1/13.9 | Affy 500 or 6.0 | DEXA |
| AOGC | 594 | 0/594 | 76.3/7.95 | 157/6 | 69/17 | illumina 370CNV Quad | DEXA |
| GOOD | 731 | 731/0 | 24.06/0.62 | 182.4/6.5 | 78.2/11.3 | illumina 610K | DEXA |
| TUK1 | 1292 | 2/1290 | 49.23/13.44 | 162.09/6.37 | 66.77/11.79 | illumina 317K | DEXA |
| TUK23 | 2226 | 296/1930 | 49.47/13.82 | 164.07/7.93 | 68.38/12.55 | illumina 610K | DEXA |
| Total | 5672 | 1432/4240 |  |  |  |  |  |
