# Supplementary material for: WNT16 Influences Bone Mineral Density, Cortical Bone Thickness, Bone Strength, and Osteoporotic Fracture Risk
Source: PLoS Genet. 2012 Jul 5;8(7):e1002745. doi: 10.1371/journal.pgen.1002745 (PMC3390364; doi:10.1371/journal.pgen.1002745)
Supplement: Table S5 — Characteristics of the included cohorts for fracture study. (DOCX) [file pgen.1002745.s015.docx]

| **Table S5.** Characteristics of the included cohorts for fracture study. | | | | | |
| --- | --- | --- | --- | --- | --- |
| Study | Sample Size | Sample Size | Percent | Age | Genotyping Platform |
|  |  | Cases/Controls | Women | mean/sd |  |
| AOGC | 1827 | 155/1672 | 100% | 69.6/8.6 | illumina 370CNV Quad |
| CaMos/ManMc | 1650 | 800/850 | 83.5% | 67.2/13.1 | Kbiosciences |
| UFO | 2286 | 1068/1218 | 79.40% | 61.0/7.3 | Kbiosciences |
| Total | 5763 | 2023/3740 |  |  |  |
