# Supplementary material for: WNT16 Influences Bone Mineral Density, Cortical Bone Thickness, Bone Strength, and Osteoporotic Fracture Risk
Source: PLoS Genet. 2012 Jul 5;8(7):e1002745. doi: 10.1371/journal.pgen.1002745 (PMC3390364; doi:10.1371/journal.pgen.1002745)
Supplement: Table S6 — Association results for the 3 fracture cohorts. (DOCX) [file pgen.1002745.s016.docx]

| **Table S6.** Association results for the 3 fracture cohorts | | | | | | |  |  |  |  |  |
| --- | --- | --- | --- | --- | --- | --- | --- | --- | --- | --- | --- |
|  |  |  |  | UFO |  |  | CaMos/ManMc |  |  | AOGC |  |
| Chr | SNP | EA | Beta | SE | P-Value | Beta | SE | P-Value | Beta | SE | P-Value |
| 7 | rs7776725 | C | -0.36 | 0.07 | 1.40E-07 | -0.21 | 0.09 | 0.018 | -0.21 | 0.14 | 0.1324 |
| 7 | rs2908004 | T | -0.19 | 0.06 | 0.001 | -0.22 | 0.08 | 0.005 | -0.21 | 0.13 | 0.0906 |
| 7 | rs2707466 | A | -0.19 | 0.06 | 0.001 | -0.21 | 0.08 | 0.006 | -0.20 | 0.13 | 0.116 |
| 7 | rs10274324 | G | -0.14 | 0.14 | 0.301 | -0.07 | 0.15 | 0.631 | -0.29 | 0.25 | 0.2452 |
| EA: effect allele | |  |  |  |  |  |  |  |  |  |  |
