# Supplementary material for: WNT16 Influences Bone Mineral Density, Cortical Bone Thickness, Bone Strength, and Osteoporotic Fracture Risk
Source: PLoS Genet. 2012 Jul 5;8(7):e1002745. doi: 10.1371/journal.pgen.1002745 (PMC3390364; doi:10.1371/journal.pgen.1002745)
Supplement: Table S7 — Micro CT parameters of the femoral shaft and fifth lumbar vertebra (LV5) in male wild type and Fam3c−/− mice. Data are presented for each of the three KO strategies and also for the combined cohorts (WT = 2; Fam3c−/− = 4 for each of the individual cohorts). (DOCX) [file pgen.1002745.s017.docx]

**Table S7.** Micro CT parameters of the femoral shaft and fifth lumbar vertebra (LV5) in male wild type and *Fam3c ^-/-^* mice. Data are presented for each of the three KO strategies and also for the combined cohorts (WT=2; *Fam3c ^-/-^* = 4 for each of the individual cohorts)

| Genotype  (KO Strategy) | LV5 Tb BV/TV (%) | Femur Shaft Total Area (mm^2^) | Femur Shaft Bone Area (mm^2^) | Femur Shaft Marrow Area (mm2) | Femur Shaft Cortical Thickness (µm) |
| --- | --- | --- | --- | --- | --- |
| WT (Gene Trap) | 20.1 ± 1.2 | 1.97 ± 0.19 | 1.06 ± 0.08 | 0.91 ± 0.11 | 259 ± 4 |
| KO (Gene Trap) | 17.5 ± 0.8 | 2.01 ± 0.14 | 1.03 ± 0.02 | 0.97 ± 0.13 | 255 ± 13 |
| WT (HR #1) | 10.7 ± 3.3 | 1.84 ± 0.31 | 0.97 ± 0.12 | 0.87 ± 0.18 | 238 ± 3 |
| KO (HR #1) | 12.0 ± 1.4 | 1.58 ± 0.15 | 0.89 ± 0.07 | 0.69 ± 0.08 | 249 ± 9 |
| WT (HR #2) | 13.6 ± 2.1 | 1.73 ± 0.01 | 0.91 ± 0.06 | 0.82 ± 0.06 | 233 ± 17 |
| KO (HR #2) | 13.3 ± 2.0 | 1.41 ± 0.11 | 0.82 ± 0.06 | 0.59 ± 0.05 | 245 ± 8 |
| WT (All Mice) | 14.8 ± 2.1 | 1.85 ± 0.10 | 0.98 ± 0.05 | 0.87 ± 0.06 | 243 ± 7 |
| KO (All Mice) | 14.3 ± 1.0 | 1.67 ± 0.10 | 0.91 ± 0.04 | 0.75 ± 0.07 | 249 ± 8 |
| KO Effect Statistics (All Mice) | ∆ = ↓4%  P = 0.79 | ∆ = ↓10%  P = 0.28 | ∆ = ↓7%  P = 0.32 | ∆ = ↓13%  P = 0.30 | ∆ = ↑2%  P = 0.52 |
